# Decoding the Identity of Pinot Gris and Pinot Noir Wines: A Comprehensive Chemometric Fusion of Sensory (from Dual Panel) and Chemical Analysis

**DOI:** 10.3390/foods13010018

**Published:** 2023-12-20

**Authors:** Aakriti Darnal, Simone Poggesi, Edoardo Longo, Annagrazia Arbore, Emanuele Boselli

**Affiliations:** 1 Faculty of Agricultural, Environmental and Food Sciences, Free University of Bozen-Bolzano, Piazza Università 1, 39100 Bolzano, Italy; aakriti.darnal@student.unibz.it (A.D.); annagraziaarbore@gmail.com (A.A.); emanuele.boselli@unibz.it (E.B.); 2 Oenolab, NOITechPark Alto Adige/Südtirol, Via A. Volta 13B, 39100 Bolzano, Italy; 3 Food Experience and Sensory Testing (Feast) Lab, Massey University, Palmerston North 4410, New Zealand; s.poggesi@massey.ac.nz

**Keywords:** MRATA (modified rate-all-that-apply) sensory analysis, HS-SPME-GCxGC-TOF/MS, HPLC-MS, Pinot Gris, Pinot Noir, cyclic proanthocyanidins, international wines

## Abstract

Quantitative relations between the sensory overall quality (OQJ) of commercial single grape variety Pinot Gris and Pinot Noir wines, defined using specific sensory attributes, and the most influencing chemical components were investigated in commercial wines from different international origins. Multiple factor analysis (MFA) was applied to achieve a comprehensive map of the quality of the samples while multivariate regression models were applied to each varietal wine to determine the sensory attributes influencing OQJ the most and to understand how the combinations of the volatile compounds influenced the olfactory sensory attributes. For Pinot Gris wine, OQJ was positively correlated with sensory attributes, like “floral” aroma, “stone-fruit” flavor, “yellow” color, “caramelized” aroma, and “tropical fruit” aroma according to an Italian panel. For Pinot Noir wine, “licorice” aroma, “cloves” aroma, “fresh wood” aroma, “red fruit” flavor, “cherry” aroma, and “spicy” flavor were positively correlated with OQJ by the same panel. Important predictors for the wine quality of Pinot Gris could be characterized, but not for Pinot Noir. Additionally, sensory tests were also carried out by different panel compositions (German and Italian). Both the German and the Italian panels preferred (based on OQJ) a Pinot Gris wine from New Zealand (Gisborne), but for different perceived characteristics (fruity and aromatic notes by the Italian panel and acidity by the German panel). For Pinot Noir, different panel compositions influenced the OQJ of the wines, as the wines from Chile (with more spicy, red fruit and woody notes) were preferred by the Italian panel, while the German panels preferred the wines from Argentina (with light, subtle woody and red fruit notes). The profile of cyclic and non-cyclic proanthocyanidins was also evaluated in the two varietal wines. No clear effect of the origin was observed, but the wines from Italy (Sicily/Puglia) were separated from the rest and were characterized by percentage ratio chemical indexes (%C-4) and (%C-5) for both varieties.

## 1. Introduction

Wines from single grape varieties but from different production areas can present different sensory attributes, as a result of the expression of the specific terroirs [1]. Terroir has been defined as a combination of soil, micro-climate, oenological technique, and grape varieties which will be expressed in the wine. As a practical consequence, wines produced in one region might be better suited for being sold in certain markets and less in others, according to local consumers’ preferences [2]. At the same time, evaluations given by the panels of wine experts, commonly employed for determining the placement of a product on the market, may still be influenced by preferences and prejudices impacting the final placement of the products on the trade [3]. Therefore, it might be relevant that this assessment is also paralleled by harmonized and standardized sensory methodologies, which might for example provide a ranking of the samples based on validated sets of criteria. As a potential additional challenge, even trained panels might be influenced by factors related to the composition of the panel itself, such as age, nationality, etc. [4]. 

Hence, further correlating the sensory analysis results to the chemical profiles might offer an additional control, obtained with an independent and standardizable set of analytical methods. This presents a double aim: (1) to represent the overall sensory results (using descriptors and OQJ, overall quality judgement) as functions of the independently determined sets of variables, and (2) to account for systematic effects inherent to the panel itself that might influence the results [5]. In a recent work, this approach was applied to single-variety international Chardonnay and Sauvignon Blanc wines; to improve such applications, the choice was made to correlate specific chemical profiles with relative subsets of the sensory attributes profile, according to the type of perception involved (e.g., visual, olfactory, and gustatory variables) [6]. However, consideration should always be taken of the fact that associating the chemical composition of the wines with the sensory properties and the wine quality is not a straightforward concept, as many volatile and non-volatile components may either contribute to or counteract the final quality, or even show no impact [7].

Indeed, sensory evaluations are influenced by interactions between different sample components (e.g., matrix effects due to the chemical composition), and between these and the perception modes. In turn, the chemical composition can be linked to the viticultural–enological variables (e.g., grape variety, winemaking technique, vintage, and terroir, just to name a few); hence, sensory analysis results could at least, in theory, be described as a complex function of the chemical composition [8,9].

The aim of this work was to characterize the chemosensory profiles of commercial single-variety Pinot Gris and Pinot Noir wines. Seven Pinot Gris and seven Pinot Noir wines from different countries (reflecting different wine styles) around the world were investigated to find out which sensory and chemical attributes were most interesting in influencing their quality. Parallelly, such attributes have been correlated to the chemical components in the wine and to the profile of cyclic and non-cyclic oligomeric proanthocyanidins. 

## 2. Materials and Methods

### 2.1. Pinot Gris and Pinot Noir Wines

Pinot Gris and Pinot Noir wine bottles were kindly provided by FruitService S.r.l., a South Tyrolean trading company (Bolzano, Italy). Each wine bottle came in triplicate and all of them were labelled with a unique three-digit code number during the experiment. Along with oenological parameters, information on their country of origin was also provided. For the Pinot Gris wines, two were produced in the Republic of South Africa, namely Western Cape (dry land) and Western Cape (irrigated area), two were produced in Italy, namely Veneto-Friuli (Northern Italy) and Puglia-Sicilia (Southern Italy), two were from New Zealand, namely Marlborough (South Island) and Gisborne/East Coast (North Island), and an additional wine came from Argentina (Mendoza). For the Pinot Noir wines, one was produced in the Republic of South Africa, namely Western Cape (dry land), two came from Italy, namely Veneto-Friuli (Northern Italy) and Puglia-Sicilia (Southern Italy), two came from New Zealand, namely Marlborough (South Island) and Hawkes Bay (North Island), one came from Argentina (Mendoza), and one came from Chile (Central Valley). Furthermore, information concerning the %vol of alcohol, the content of residual sugars, titratable acidity, and pH are also reported in Table 1 and Table 2.

### 2.2. Climate of the Geographical Area

In Table 3, climate details for the different origins of the samples are given. 

### 2.3. Sensory Analysis

For the sensory analysis of Pinot Gris and Pinot Noir wines, three phases (defining the sensory attributes, rapid training, and RATA test) each consisting of one round table, two training sessions (1 h each), and two MRATA sessions (modified rate-all-that-apply [6,21]) were performed. The sessions were held on different days for the different varieties. Cysensy, an SQL binding sensory analysis web software developed in collaboration with the Computer Science faculty of the Free University of Bozen/Bolzano, was used during the training and the MRATA sessions.

The panelists were recruited on a voluntary basis and included students and staff of the Free University of Bozen/Bolzano (Italy). All panelists voluntarily agreed to participate and signed an informed consent form, which was obtained from all individual participants included in the sensory study. Training and sensory testing were conducted in the same room and at the same time for one month. To limit the impact of location and time on the reproducibility of the sensory analysis, these parameters were maintained for all sessions. The maximum quantity of alcohol/session was 32 mL/session, so the study was considered low risk for two reasons: the participants were instructed to spit the wine after the analysis, and the sample were commercial products available on the market. For these reasons, approval from an ethics committee was not required. 

The panelists for both Pinot Gris (ten people, 40% males, 60% females, aged between 25 ± 2 years old) and Pinot Noir (eleven people, 55% females, 45% males, 25 ± 2 years old) were recruited based on their interest in the project. In the round table session, they were asked to test each wine individually and to share descriptors of visual, olfactory, and gustatory (taste and flavor) attributes on an interactive web whiteboard (Jamboard, Google, Dublin, Ireland). After each sample, the panel leader and panelists discussed and selected the most appropriate shared attribute for each wine type, to create the final sensory lexicon.

Then, the panelists were trained for those specific attributes (shown in Table 4 and Table 5). For both varieties, the first training session was specific to the odor attributes. Panelists were trained with reference standards [22] or natural standards, if references were not available. The second training session was assigned to taste and flavor attributes along with those odor attributes that the panelists did not recognize at a percentage higher than 75% during the descriptor test of the first training session. A third training session was required for Pinot Noir, in which panelists were trained on flavors, on the remaining taste attributes (sweetness, warmness), and on two aromas not successfully recognized during the previous training sessions. Moreover, in addition to the two new taste attributes, panelists were further trained for astringency. The results of the training sessions are reported in Supplementary File S1—Tables S1–S4 for Pinot Gris and Tables S5–S10 for Pinot Noir.

Then, the panelists performed a modified RATA test in which they were asked to rate the intensity of each attribute from 0 to 5, where 0 was “does not apply”, 1 was “weak” and 5 was “strong”. 

The wine samples were analyzed in duplicate in two different MRATA sessions. The bottles were opened 30 min before the session. An aliquot (30 mL) of wine was poured into ISO glasses labelled with a random three-digit number. The tasting order of the wine samples was randomized using a complete Latin square balance design among the panelists to reduce carry-over effects [23,24]. In addition, a sensory test was also performed by local German panel in an accredited laboratory for wine testing (DIN EN ISO/IEC 17025:2018). The analysis results were provided by the company. 

### 2.4. HS-SPME-GCxGC-ToF/MS Analysis of the Volatile Profiles in Pinot Gris and Pinot Noir Wines

The method applied was slightly adapted from published reports [6]. Each sample was prepared and analyzed in GCxGC at the same time after the sampling (bottle opening) to minimize sampling errors. For each sample preparation, 0.5 g of NaCl was placed into a 10 mL SPME glass vial. Then, 4 mL of wine sample was added to the vial. Next, 10 µL of 2-methyl-3-pentanol was added as an internal standard (from a stock of 1/50 of I.S. in ethanol). Then, the vial was closed with a perforable screwcap with a silicon septum for automated SPME. The SPME was performed with an LPAL3 GC autosampler equipped with a Peltier stack, where the samples were kept at 4° C before the analysis. For SPME extraction, a Stableflex SPME fiber 50/30 µm DVB/CAR/PDMS, 23 Ga, for the autosampler (SUPELCO, 595 North Harrison Road, Bellefonte, PA 16823-0048, USA) was used. The parameters applied for the SPME sample extraction were: (1) sample incubation at 40 °C for 15 min with 300 rpm agitation (5 s on time, 2 s off time), (2) sample extraction for 30 min (fiber penetration depth in the vial = 25 mm), and (3) sample injection with 6 min desorbing time in the split/splitless inlet of the GC (fiber penetration depth = 40 mm). 

The fiber was preconditioned at 240 °C for 6 min before analyzing each sample. The separation was performed by comprehensive GCxGC on an instrument coupled with a Pegasus BT 4D time-of-flight mass spectrometer, and equipped with FluxTM flow modulator (Leco Italy, Milano). The separation was carried out at 1 mL min^−1^ (He carrier gas) in splitless mode, with 2 mL min^−1^ septum purge flow and 6 mL min^−1^ inlet purge flow. The inlet temperature was 240 °C. The separation was carried in GCxGC on a polar cross-bond PEG-phase MEGA-Wax Spirit 0.30 µm × 0.18 mm × 40 m (Mega S.r.l., Legnano, Italy) as the first dimension and a Rxi-17 Sil 0.10 µm × 0.10 mm × (0.7 + 0.31) mm (Restek S.r.l., Cernusco sul Naviglio, Italy) as the second dimension. The two columns were connected by a FluxTM modulator (LECO), with a 15.92 psi auxiliary diverting helium flow. The second column was mounted in a secondary oven inside (but thermally isolated from) the larger primary oven. The primary oven temperature program was 40 °C for 6 min, 40 to 180 °C at 3 °C min^−1^, 180 to 240 °C at 10 °C min^−1^, and 240 °C for 1 min. The secondary oven had a temperature 5 °C higher than the first oven, and, accordingly, its temperature program was shifted to that of the same temperature difference. The GCxGC instrument was connected to the mass spectrometer by a transfer line kept at 250 °C. The mass spectrometer operated with the following parameters: 0 s acquisition delay, 70 eV filament electron energy, 250 °C ion source temperature, 150 spectra/s acquisition rate, 32 kHz extraction frequency, and acquisition mass range = *m/z* 35–530. The mass spectrometer was tuned every time before starting a new sequence of analyses. After each analysis, each GCxGC chromatogram was reconstructed in 2D color maps by ChromaTOF^®^ (LECO Corporation, Berlin, Germany) ver.2021 software by deconvolution. Automatic compound assignment was carried out by NIST 2017 (NIST MS Search 2.3) database comparison. Linear retention indexes were calculated from the first-dimension retention times against the injected series of linear fatty ethyl esters C4-C24 (even carbon saturated, Merk Life S.r.l., Milano, Italy).

The collected dataset underwent automatic alignment with the use of the processing software ChromaTOF^®^ (LECO Corporation, Berlin, Germany) ver. 2021. The obtained aligned table was inspected and refined prior to the statistical analysis. 

### 2.5. UHPLC-MS Analysis of the Non-Anthocyanic Phenolic Profiles in Pinot Noir and Pinot Gris Wines

The applied method was slightly adapted from published reports [6]. The LC-MS analysis of the non-anthocyanin polyphenols has been carried out on a UHPLC-QqQ/MS instrument (Agilent LC/TQ 6465 system) equipped with a 1260 Infinity II UHPLC with a quaternary pumps system, a 1260 Infinity II WR PDA detector, in series with an AJS ESI QqQ mass analyzer. The chromatographic separations have been carried out on a Poroshell 120, SB-C18 2.1 mm × 100 mm × 2.7 µm (Agilent Technologies Italia, Milan) kept at 30 °C at a 0.35 mL/min flow rate. The mobile phase consisted of (A) 0.1% formic acid in degassed ultrapure water and (B) 0.1% formic acid in acetonitrile. All used solvents and mobile phase additives were at MS grade. The gradient separation program was as follows: 1%B from 0 to 1.5 min, 1 to 30% B from 1.5 to 19 min, 30 to 99% B from 19 to 20 min, 99% B from 20 to 24 min, 99 to 1% B from 24 to 25 min, and 1% B from 25 to 30 min. The injection volume was 5 µL. The PDA detector was set to record the absorbance in the 200–700 nm wavelength range using a 4 s response time (1.25 Hz) and 4 nm slit width, with 1 nm spectrum steps. The MS detection was carried in ESI- ionization mode, with the following parameters: mass range = *m/z* 200–750, scan time = 500, step size = 0.1 amu, fragmentor potential = 135 V, cell acceleration = 5 V, N_2_ gas temperature = 260 °C, N_2_ gas flow = 4 L/min, nebulizer pressure = 35 psi, sheath gas heater = 300 °C, sheath gas flow = 12 L/min, capillary voltage = −2500 V, and nozzle voltage = −500 V. 

Tentative compound identification was carried out by full-scan mass spectrometry determination and the relative PDA λmax assignment, to classify the compounds at least in a phenolic class, where a complete identification could not be achieved. Solutions of standard compounds (gallic acid, trans-caffeic acid, p-coumaric acid, trans-caftaric acid, (+)-catechin, (−)-epicatechin, protocatechuic acid, astilbin, kaempferol-3-O-glucoside, and procyanidin B2) were analyzed by standard injection, and their PDA spectra, MS/MS spectra, and retention times (min) were used as references. Targeted MS/MS fragmentation experiments (product ions monitoring—PRM) on selected ions were carried out with the following setup: source parameters as in the MS1 analysis, acquisition mass range from *m/z* 25 to + 10 *m/z* from the selected precursor ion, scan time = 125 ms, fragmentor potential = 135 V, collision energy = 25 V, and cell accelerator 5 V. 

Oligomeric proanthocyanidins (PAC) were separately analyzed on the same UHPLC-QqQ/MS instrument (Agilent LC/TQ 6465 system). The chromatographic separation was carried out on a Vertex Plus Eurosphere II (KNAUER, Berlin, Germany) column (4.6 mm × 250 mm × 5 µm) with a precolumn, and kept at 30 °C. The separation was carried out at a 0.7 mL/min flow rate. The mobile phases consisted of (A) 0.1% formic acid in degassed ultrapure water and (B) 0.1% formic acid in acetonitrile. All solvents and mobile phase additives were at MS grade. The gradient separation program was 1%B from 0 to 2.5 min, 1 to 25%B from 2.5 to 50 min, 25 to 99%B from 50 to 51 min, 99%B from 51 to 55 min, 99 to 1%B from 55 to 56 min, and 1%B from 56 to 62 min. The injection volume was 5 µL. The mass spectrometer operated in ESI+ mode. The detection of the proanthocyanidins was performed in single-ion monitoring (SIM) mode. In Supplementary File S1—Table S14, the targeted SIM parameters for acquisition for both wines are listed. The parameters for mass spectrometric detection were as follows: fragmentor = 135, cell acceleration = 5 V, N_2_ gas temperature = 230 °C, N_2_ gas flow = 8 L/min, nebulizer pressure = 20 psi, sheath gas heater = 300 °C, sheath gas flow = 10 L/min, capillary voltage = 3000 V, and nozzle voltage = 2000 V. Feature selection (retention time/*m/z* ratio) took place by directly inspecting the acquired data, and these selected features were applied to directly extract the peak intensities in MzMine3 and create the peak table. Multiple peaks at the same retention time were double-checked for the presence of in-source formed adducts and fragments by analysis of the peak list’s correlation matrix (applied criteria for filtering: 1—a DRt between features ≤ 0.1 min; 2—significant correlation between two features; 3—an rPearson ≥ + 0.75). 

### 2.6. UHPLC-MS Analysis of the Anthocyanic Phenolic Profiles in Pinot Noir Wines

LC-MS parameters for the analysis of anthocyanins of Pinot Noir were as follows: injection volume = 5 µL; mobile phase composition, phase A = 4.5% formic acid degassed in ultrapure water; phase B = 4.5% formic acid in MS-grade acetonitrile; the gradient program was as follows: 5% B from 0 to 1 min, 5–15% B from 1 to 10 min, 15 to 25%B from 10 to 15 min, 25 to 40%B from 15 to 18 min, 40 to 95% from 18 to 21 min, 95% from 21 to 24 min, 95 to 5% from 24 to 25 min, and 5% from 25 to 28 min. The ESI source was run in positive mode with the following parameters: gas temperature 340 °C, nitrogen gas flow = 13 L min^−1^, nebulizer pressure 50 psi, nitrogen sheath gas heater = 350 °C, sheath gas flow = 12 L min^−1^, capillary voltage +3500 V, and nozzle voltage = + 1000 V. ESI-MS parameters for anthocyanins were as follows: mass range = *m/z* 250–700, scan time = 500, size fragmentor potential = 135 V, and cell acceleration voltage = 5 V. DAD detector parameters were as follows: wavelength 1 range = 700–210 nm, response time = 4 s, acq. frequency = 1.25 Hz and slit width = 4 nm (with 1 nm spectrum steps). For anthocyanins in Pinot Noir, at first a targeted selection was run, selecting only those features with an absorbance band between 450 and 550 nm (Supplementary File S1—Table S17). Then, the corresponding MS traces were identified, looking for the precursor ions and for specific in-source fragments, e.g., *m/z* 493 and *m/z* 331 for malvidin-3-O-glucoside and its aglycone, respectively. An example for the targeted analysis of peonidin-3-O-glucoside (*m/z* 463 and *m/z* 301) is represented in the Supplementary File S1—Figure S1.

The relative concentrations (mg.L ^−1^) of anthocyanins in the Pinot Noir samples were calculated against a calibration curve of the reference standard of malvidin chloride (MvCl) prepared at different concentrations (0.0, 0.05, 0.2, 0.5, and 1.0 gL^−1^) in duplicates.

### 2.7. Statistical Analysis 

The statistical analysis was performed with the XLSTAT add-on for Excel (Lumivero, Denver, CO, USA). The same statistical methods applied in a recently published report have been applied [6]. One-way analysis of variance (ANOVA) followed by the Tukey’s HSD (honestly significant difference) post hoc test was applied. The whole datasets were investigated by agglomerative hierarchical clustering (AHC) method, have been computed using dissimilarity and Euclidean distance coefficient and have been explained briefly. Multiple factor analysis (MFA) was also applied as an explorative tool to analyze all datasets, which contained different groups of variables (both quantitative and qualitative) [25,26], in this study, all of which are quantitative. 

Partial least-squares regression (PLS-R) has been used to correlate the sensory descriptor variables with the overall quality judgment to understand which descriptors most affected the quality of the product. Additionally, the method was also used to identify which volatile compounds affected the olfactory sensory attributes [6]. 

## 3. Results

In the first part of this section, ANOVA (for significant sensory variables) and explorative methods, such as AHC and MFA for the whole dataset including both chemical and sensory parameters, are reported. The list of identified volatile compounds in Pinot Gris and Pinot Noir are reported in the Supplementary File S1—Tables S11 and S12. The tentative identification of phenolic compounds is reported in Supplementary File S1—Table S13, and the fragmentation experiments are reported in Supplementary File S2. The list of observed proanthocyanidins is reported in Supplementary File S1—Tables S15 and S16 for Pinot Gris and Pinot Noir, respectively. Additionally, percentage ratios (%) of the cyclic tetrameric procyanidin (%C-4), cyclic tetrameric prodelphinidin with one (epi)gallocatechin and three (epi)catechins (%C-4-OH), and cyclic pentameric procyanidin (%C-5) were calculated as a concentration-normalized parameter (equation below [27]) that have been proposed in the previous literature as a wine authenticity parameter and here have been applied to investigate for the different wine origins [6,28]. Equation (1) is as follows:

%-ratio = 100 × [cyclic]/([cyclic] + [non-cyclic])(1)

where [cyclic] represents the relative abundance of a specific cyclic PAC (e.g., cyclic B-type tetrameric procyanidin, ESI+ *m/z* 1153), and [non-cyclic] is the sum of all the relative abundances in the SIM trace of all those isobaric and isomeric species having the same degree of polymerization (DP), as the cyclic species and the same monomeric composition (in terms of the proportion of (epi)catechins and (epi)gallocatechins, but being conventional B-type proanthocyanins, therefore, differing by +2 Da from their own cyclic analogue (e.g., all B-type tetrameric procyanidins isomers, ESI+ *m/z* 1155).

The second part of the section deals with the regression models for both varieties. The datasets were separated rationally, with sensory attributes vs. OQJ followed by aroma compounds vs. olfactory sensory attributes. Lastly, a spider plot for the comparison of the overall quality scores provided by two different panels (German and Italian) is reported. 

### 3.1. ANOVA

#### 3.1.1. Pinot Gris Sensory Data 

**Figure 1:**
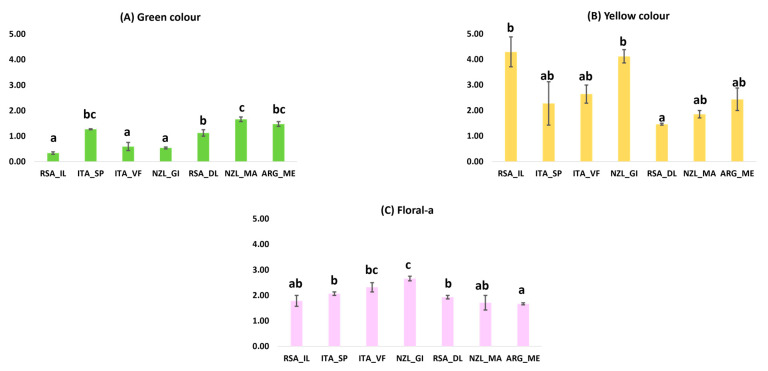
(**A**–**C**) Histograms for significantly different attributes for the origin of Pinot Gris, with the groupings indicated by the letters that were obtained with Tukey’s HSD post hoc test (*p* < 0.05) reported with standard deviations.

In Figure 1A–C, the averaged results obtained for the significant (one-way ANOVA, *p*-val. < 0.05) sensory attributes with respect to different origins are illustrated. These were “green” color, “yellow” color, and “floral” aroma. For the “green” color, the samples obtaining the lowest score were RSA_IL, followed by ITA_VF and NZL_GI (belonging to the same significance group). NZL_MA samples received the highest score for “green” color intensity. Conversely, “yellow” color intensity resulted the highest for RSA_IL and NZL_GI samples, showing high variability for RSA_IL. The score for the lowest “yellow” color intensity for this attribute was instead associated to RSA_DL samples. For “floral” aroma, the highest score was assigned to NZL_GI wines, and the lowest was assigned to ARG_ME wines. RSA_DL and ITA_SP constituted an intermediate but separate group on their own.

#### 3.1.2. Pinot Noir Sensory Data 

**Figure 2:**
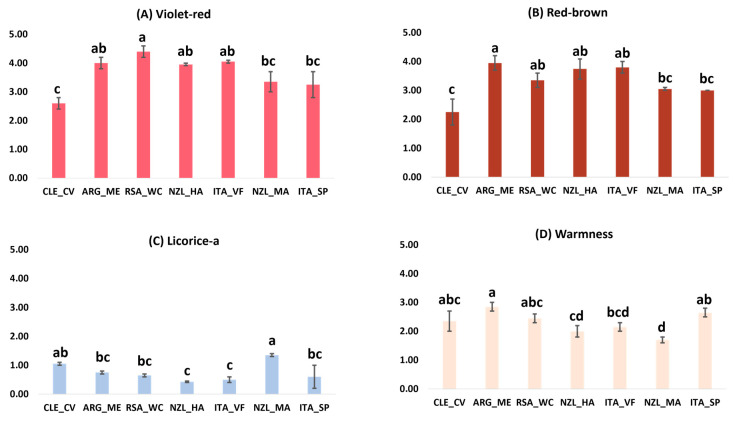
(**A**–**D**) Histograms for significantly different attributes for the origin of Pinot Noir, with the groupings indicated by the letters that were obtained with Tukey’s HSD post hoc test (*p* < 0.05) reported with standard deviations.

In Figure 2A–D, the averaged results are indicated for each wine category. Significance (1-way ANOVA, *p*-val. < 0.05) was calculated across wine origins. The sensory attributes “violet-red” color, “red-brown” color, “licorice” aroma, and “warmness” were significantly different across the samples. For “violet-red” color, the sample obtaining the lowest score was CLE_CV, followed by NZL_MA and ITA_SP (belonging to the same significance group), while the sample that obtained the highest score was RSA_WC. Again, the sample with the lowest “red-brown color” score was CLE_CV, while the sample with the highest score was ARG_ME. The average score for “licorice” aroma was comparatively lower than other significant attributes for Pinot Noir. Interestingly, the highest and the lowest score for this attribute was assigned to the wines both originating from New Zealand, with the highest score given to NZL_MA and the lowest to NZL_HA. Lastly, for “warmness” attribute, the highest score was associated to ARG_ME and the lowest to NZL_MA.

### 3.2. Agglomerative Hierarchical Clustering (AHC)

**Figure 3:**
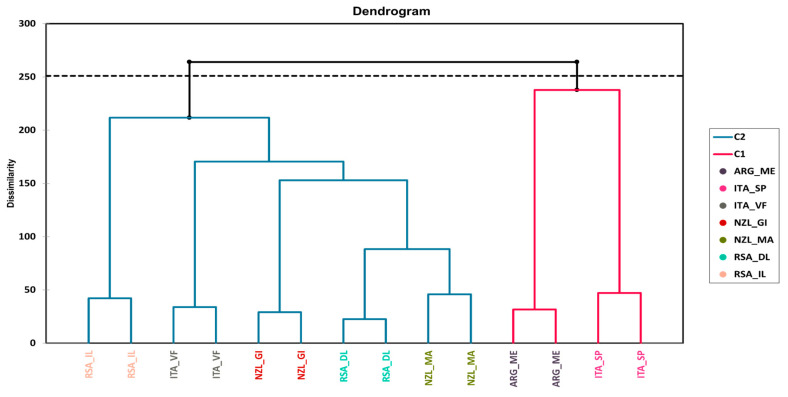
Dendrogram representing the agglomerative hierarchical clustering (AHC) on all the datasets (both chemical and sensory) of Pinot Gris wines from different origins. The Euclidean distance was used for the dissimilarity scale and Ward’s method for agglomeration. The dotted line represents the automatic truncation based on entropy.

**Figure 4:**
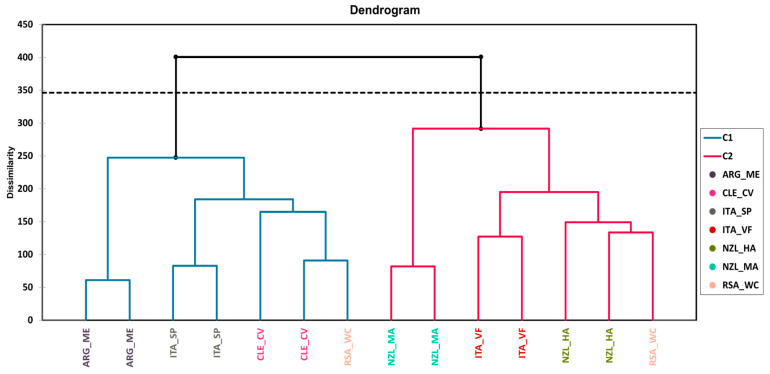
Dendrogram representing the agglomerative hierarchical clustering (AHC) on all the datasets of Pinot Noir wines from different origins. The Euclidean distance was used for the dissimilarity scale and Ward’s method for agglomeration. The dotted line represents the automatic truncation based on entropy.

The results for AHC are only concisely discussed here, as the data are more exhaustively explained by the MFA in the next paragraph. The dendrograms representing the AHC based on the dissimilarities using Ward’s method on all the datasets (both chemical and sensory) used for the MFA are presented in Figure 3 for Pinot Gris wine samples and in Figure 4 for Pinot Noir wine samples. 

AHC was able to successfully group all the observations for Pinot Gris wines (Figure 3). The first cluster grouped the analyzed wines from Argentina and from Italy (Sicily/Puglia) while the second cluster grouped the rest of the wines (from South Africa, New Zealand, and Veneto/Friuli—Italy). 

In the case of Pinot Noir, AHC also formed two clusters (Figure 4). The first cluster grouped the analyzed wines from Argentina and analyzed wines from Italy (Sicily/Puglia), like for Pinot Gris. However, the analyzed wines from Chile were also grouped in this cluster, while the second cluster grouped the rest of the wines. The wine replicates from South Africa (Western Cape, dry land) formed different clusters, where one of the replicates showed more similarity with the wines from Chile in the first cluster, while the other replicate showed similarity with the wines from New Zealand (Hawkes Bay). 

Multiple factor analysis and multivariate modelling were also used (see the following paragraphs) to determine which chemical variables were responsible for these observed separations.

### 3.3. Multiple Factor Analysis (MFA)

#### 3.3.1. Pinot Gris Wines

**Figure 5:**
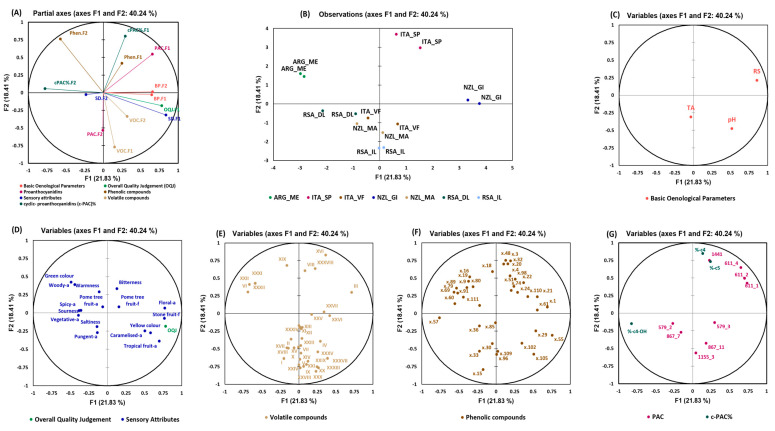
MFA for the Pinot Gris dataset. (**A**) The partial axes for the first 3 components and the different datasets used in the computation; (**B**) observation plot and (**C**–**G**) basic oenological variables (3), sensory analysis variables (17), volatile compounds (37), phenolic compounds (37), and proanthocyanidins (9)/c-PAC% (3), respectively. Identified volatile compounds: I = ethyl acetate, II = ethyl butanoate, III = isobutyl alcohol, IV = isoamyl acetate, V = ethyl hexanoate, VI = isopentanol, VII = hexyl acetate, VIII = 3- ethyl-3-hexanol, IX = ethyl octanoate, X = acetic acid, XI = ethyl sorbate (isomer I), XII = ethyl sorbate (isomer II), XIII = benzaldehyde, XIV = nonanoic acid, 2-oxo-, methyl ester, XV = octanoic, phenyl ester, XVI = linalool, XVII = 2,3-butanediol (I), XVIII = 2,3-butanediol (II), XIX = ethyl 2-furoate, XX = ethyl decanoate, XXI = isopentyl octanoate, XXII = ethyl succinate, XXIII = α- terpineol, XXIV = vinyl decanoate, XXV = ethyl phenylacetate, XXVI = 2-phenethyl acetate, XXVII= benzenebutanal, XXVIII = ethyl dodecanoate, XXIX = hexanoic acid, XXX = isoamyl decanoate, XXXI = ethyl isopentyl succinate, XXXII = phenylethyl alcohol, XXXIII = octanoic acid, XXXIV = sorbic acid, XXXV = ethyl hexadecanoate, XXXVII = decanoic acid, and XXXVIII = 2,4-di-tert-butylphenol; RS, residual sugars; TA, titratable acidity.

In Figure 5, the MFA model for Pinot Gris wines is presented, which includes all datasets joined in a unique model not influenced by the shear numerosity of the variables within the specific datasets themselves (as it would with PCA). In this case, these datasets are as follows: basic oenological parameters, sensory attributes, volatile compounds, phenolic compounds, and proanthocyanidins. The results of the separate PCA conducted on each dataset allow for better understanding of the relationship between the observations and the variables. Figure 5A represents the projection onto the partial axes space representing a total variance of 40%. From the observations plot in Figure 5B, the analyzed wines from Argentina, South Africa (dry land), and New Zealand (Gisborne) were well separated along F1; however, analyzed wines from Italy (Sicily/Puglia), New Zealand (Marlborough), Italy (Veneto/Friuli), and South Africa (irrigated land) were close to each other and separated mostly in the F2. It can be clearly observed that the analyzed wines from Italy (Sicily/Puglia) were mostly separated due to the contribution of phenolic compounds, proanthocyanidins—F1, and % ratios of cyclic proanthocyanidins (c-PAC%)—F1. Similarly, phenolic compounds—F2 contributed to the separation of the analyzed wines from Argentina; c-PAC%—F2 and sensory attributes—F2 contributed to the separation of the wines from South Africa (dry land). The wines from New Zealand were separated due to the contribution of basic oenological parameters—F2 and —F1, sensory attributes—F1, and the overall quality judgement (OQJ). Wines from Veneto/Friuli (Italy) were separated due to volatile compounds—F1, and the rest of the analyzed wines from South African from irrigated land were separated due to the contribution of volatile compounds—F1 and proanthocyanidins—F2. 

The residual sugars were the highest in the analyzed wines from New Zealand (Gisborne) and showed high correlation with these wines in Figure 5C. The total titratable acidity, on the other hand, was highly correlated with the analyzed wines from Italy (Veneto/Friuli) and New Zealand (Marlborough). In terms of the sensory attributes, the analyzed Argentinian wine samples were characterized by “green” color, “woody” aroma, and “warmness”, and showed anti-correlation with the OQJ while the wines from New Zealand (Gisborne) were strongly correlated with the OQJ, and were characterized by attributes, such as “floral” aroma, “stone-fruit” flavor, “yellow” color, “caramelized” aroma, and “tropical fruit” aroma. The volatile compounds that characterized the analyzed wines from New Zealand (Gisborne) were III: isobutyl alcohol, responsible for alcohol, pungent, solvent, or bitter aromas [29], XXVII: benzenebutanal, XXV: ethyl phenylacetate, esters responsible for fruit, sweet odors [30], and XXVI: phenethyl acetate, known for the contribution of fruity notes in wine [31]. 

In Figure 5F, the correlations of the distributions of the phenolic compounds with specific samples are shown. Only the compounds that show a higher correlation with the overall quality are highlighted. The full MS and fragmentations of the phenolic compounds from our analysis were compared with the current literature reports and, hence, were identified tentatively. The ions x57 (*m/z* 157 at 6.7 min), x60 (*m/z* 259 at 6.8 min), x79 (*m/z* 175.1 at 7.6 min), x89 (*m/z* 113 at 9.2 min), and x91 (*m/z* 177 at 9.5 min) contributed in the separation of the analyzed Argentinian wines from other origins and showed a negative correlation with the OQJ, while ions x1 (*m/z* 193 at 1.1 min), x29 (*m/z* 391.1 at 4.3 min) x55 (*m/z* 315.1 at 6.6 min), x61 (*m/z* 311.1 at 6.9 min), x102 (*m/z* 435.3 at 12.4 min), and x105 (*m/z* 449.2 at 14.2 min) showed a higher contribution in the separation of the analyzed wines from New Zealand (Gisborne) and were positively correlated with the OQJ. 

The ion x57 was characterized by *m/z* 157 (at 6.7 min), which was observed as an in-source fragment of *m/z* 461, which was a co-eluting peak, and, therefore, it was tentatively assigned to kaempferol glucuronide [32]. Ion x79 (*m/z* 175.1 at 7.6 min) has also been reported by Poggesi et al., 2022, as a fragment ion of *m/z* 373.1, whose structure could not be identified and showed a similar fragmentation pattern with ascorbic acid (*m/z* 175 and *m/z* 157 [6]). x89 at *m/z* 113 with a retention time of 9.2 min showed a precursor ion at *m/z* 289 co-eluting with it in full MS mode. Hence, this compound could be an in-source fragment of catechin [33]. Furthermore, ion x91 was identified as a phenolic moiety of aesculetin (*m/z* 177 at 9.5 min) [34,35]. Ion x29 was characterized by *m/z* 391.1 at 4.3 min and was hypothesized to be an adduct of sulfonated caftaric acid, as it showed the same MS/MS pattern (*m/z* 259, *m/z* 241, *m/z* 213, *m/z* 161, and *m/z* 149) as reported by Hayasaka et al., 2017 [36]. Additionally, ion x55, which was characterized by *m/z* 315.1 at 6.6 min, was identified as a glycosylated derivative of dihydroxybenzoic acid, but the presence of MS^2^ *m/z* 123 excluded the hypothesis that it is a protocatechuic derivative [6,32]. Ion x61 (*m/z* 311.1 at 6.9 min) was identified as caftaric acid, as also reported by Poggesi et al., 2022, and was characterized by *m/z* 149 and *m/z* 135 MS^2^ [6]. The x105 species at *m/z* 449.2 eluting at 14.2 min was identified as astilbin, also in agreement with some previously published reports from the same research group [37,38]. 

The proanthocyanidins (PACs) that distinguished the analyzed wines from New Zealand were the (non-cyclic) prodelphinidin dimers (*m/z* 611), while the Italian wine samples from Sicily/Puglia were characteristic of a cyclic pentamer (*m/z* 1441). The associated % ratios were also calculated for cyclic tetrameric procyanidin (%C-4), cyclic tetrameric prodelphinidin with one (epi)gallocatechin and three (epi)catechins (%C-4-OH), and cyclic pentameric procyanidin (%C-5) [6,27]. The analyzed wines from Italy (Sicily/Puglia) were characterized by higher percentage ratios (%C-4) and (%C-5) than the rest of the wines. The phenolic compounds that characterized these wines were ions x18 (*m/z* 251 at 2.4 min), x20 (*m/z* 347 at 2.7 min), x32 (*m/z* 153 at 4.7 min), x48 (*m/z* at 419 at 5.9 min), and x98 (*m/z* 197 at 11.5 min) which showed a positive correlation with these % ratios. These compounds have also been tentatively identified. Ion x18 with *m/z* 251 at 2.4 min was identified as a dimer of ethyl sulfate which was evident when looking at its MS/MS fragmentation spectra at 25 eV of *m/z* 125 and *m/z* 97, respectively [39]. Ion x32 was characterized by precursor ion *m/z* 153 and a fragment ion *m/z* 109 in the product ion mode. It was speculated to be a protocatechuic acid hexoside derivative [6]. Ion x98 was tentatively identified as ethyl gallate which loses an ethyl unit and a gallic unit and then a CO_2_ group with the precursor ion *m/z* 197 at 11.5 min and its characteristic fragment ions at *m/z* 169 and at *m/z* 125 [40].

#### 3.3.2. Pinot Noir Wines

**Figure 6:**
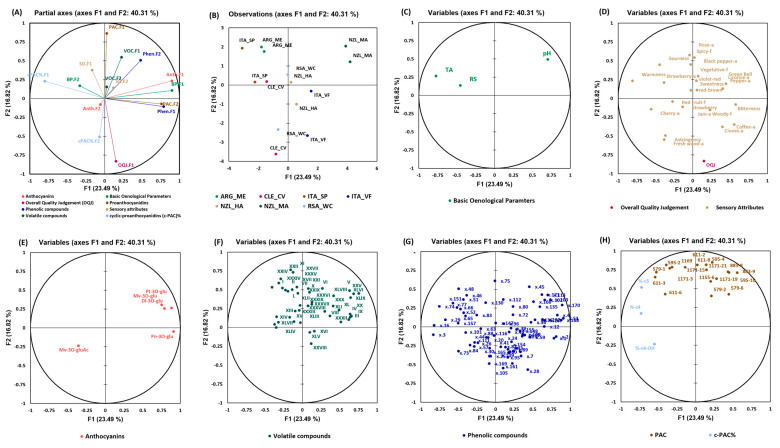
MFA for the Pinot Noir dataset. (**A**) Partial axes for the first 3 components and the different datasets used in the computation, (**B**) observation plot, and (**C**–**H**) basic oenological variables (3), sensory analysis variables (22), anthocyanins (5), volatile compounds (44), phenolic compounds (75), and proanthocyanidins (18)/c-PAC% (3), respectively. Identified volatile compounds: I = ethyl acetate, II = isoamyl acetate, III = isopentanol, IV = ethyl hexanoate, V = hexyl acetate, VI = furfuryl ethyl ester, VII = ethyl lactate, VIII = 1-hexanol, IX = ethyl octanoate, X = furfural, XI = acetic acid, XIII = 2-ethyl-1-hexanol, XIV = ethyl sorbate, XV = 2(1H)-naphthalenone, 3,4,4a,5,6,7-hexahydro-1,1,4a-trimethyl-, XVI = benzaldehyde, XVII = β-ionone, XX = linalyl acetate, XXI = isoamyl lactate, XXII = 2,3-butanediol, XXIV = ethyl furoate, XXV = ethyl decanoate, XXVII = ethyl succinate, XXVIII = ethyl 9-decenoate, XXIX = α-terpineol, XXX = 4-tert-butylcyclohexanol, XXXII = z-9-tetradecenyl acetate, XXXIII = ethyl phenylacetate, XXXVIII = trans-cubebol, XXXV = 2-phenethyl acetate, XXXVI = 4-methyl-6-phenyltetrahydro-1,3-oxazine-2-thione, XXXVII = benzenebutanal, XXXVIII = β- damascenone, XXXIX = cyclobuta[a]dibenzo[c,f]cycloheptadiene,7-oxo-, XL = hexanoic acid, XLI = benzyl alcohol, XLII = trans-whiskey lactone, XLIII = ethyl isopentyl succinate, XLIV = 4-ethylguaiacol, XLV = nerolidol, XLVI = octanoic acid, XLVII = 4-ethylphenol, and XLVIII = ethyl hexadecanoate, XLIX = n-decanoic acid, L = 2,4-di-tert-butylphenol; RS, residual sugars; TA, titratable acidity.

Figure 6 shows the MFA for Pinot Noir wines that were computed using the datasets of basic oenological parameters, sensory attributes, volatile compounds, phenolic compounds, proanthocyanidins, and anthocyanins to obtain an integrated picture of the observations and of the relationships between the different groups of variables. The partial axis built on the first two components is shown in Figure 6A with a total variance of 40.3%. It can be observed that the wines were not clearly separated, as some wine replicates were far from each other (Figure 6B). However, the separation of the analyzed wines from New Zealand (Marlborough) was due to the contribution of phenolic—F2 and anthocyanin—F1. Analyzed wines from Chile were separated due to the contribution of OQJ—F1, while the wines from Italy (Sicily/Puglia) were separated due to c-PAC%—F1 and basic oenological parameters—F2. The analyzed Argentinian wines were mostly separated due to the contribution of sensory attributes—F1. c-PAC%—F2 mainly separated the South African (Western Cape, dry land) wines. The analyzed wines from New Zealand (Hawkes Bay) were separated due to the contribution of volatile compounds—F2 and sensory attributes—F2.

From Figure 6B,C, a correlation between pH and the analyzed wines from New Zealand (Marlborough) can be clearly observed and was indeed the highest in these wines. The content of residual sugars, on the other hand, characterized the wines from Italy (Sicily/Puglia) and was the highest in these wines. From Figure 6B,D, correlation among the sensory variables and the samples can be observed. The OQJ was positively correlated with the analyzed Chilean wines that were characterized by sensory attributes, such as “licorice” aroma, “cloves” aroma, “fresh wood” aroma, “red fruit” flavor, “cherry” aroma, and “spicy” flavor. The wines that were negatively correlated with the OQJ were the analyzed Argentinian wines with sensory attributes, such as “sourness”, “rose” aroma, and “spicy” flavor. The anthocyanins that characterized the analyzed wines from New Zealand (Marlborough) were petunidin-3-O-glucoside, malvidin-3-O-glucoside, and delphinidin-3-O-glucoside. The Chilean wines were more characteristic of malvidin-3-O-(6’-acetyl)-glucoside) (Figure 6B,E). 

The analyzed wines from Chile were also characterized by the following volatile compounds (Figure 6F): II: isoamyl acetate, known to be responsible for tropical/banana odor in wine [41,42], XIII: 2-ethyl-1-hexanol, known for sweet floral odor [43], XIV: ethyl sorbate, XXXV: phenethyl acetate, known for flowery notes [44], and XLVII: 4-ethylphenol usually associated with Brett off-odor, which have been shown to increase plastic odors and decrease fruit odors in wines [45]. Indeed, the sensory panel reported off-odors, like horse and leather, in these wine samples. 

Only the phenolic compounds that showed a correlation with the overall quality are highlighted for Pinot Noir wines as well. The full MS and fragmentations of the phenolic compounds from our analysis were compared with the current literature reports and, hence, were identified tentatively. x29 (*m/z* 265.1 at 1.8 min), x57 (*m/z* 345.2 at 4.3 min), x84 (*m/z* 315.2 at 6.6 min), x101 (*m/z* 341.2 at 8.9 min), x151 (*m/z* 287.1 at 13.9 min), and x157 (*m/z* 161 at 14.4 min) were the phenolic compounds that characterized the analyzed wines from Chile and were positively correlated with the OQJ, while the ions x46 (*m/z* 191.0 at 2.9 min), x48 (*m/z* 205.0 at 2.9 min), x75 (*m/z* 417.0 at 6.0 min), and x114 (*m/z* 357.0 at 9.6 min) were negatively correlated with the OQJ, and characterized the analyzed wines from Argentina.

Ion x57 was characterized by *m/z* 345.2, which showed the fragment ion *m/z* 161 and was speculated to be the β-D-glucopyranoside precursor of guaiacol with a similar fragmentation pattern. However, fragment ions *m/z* 285.1 and *m/z* 123 were not found in our mass spectrum, most likely due to their strong fragmentation potential [46]. x84 was characterized by *m/z* 315.2 eluting at the same retention time of 6.6 min, which was also present in the Pinot Gris wines and was identified to be a glycosylated derivative of dihydroxybenzoic acid [32]. The x101 peak was identified as caffeic acid hexoside and was characterized by *m/z* 341.2 at 8.9 min which exhibited its MS^2^ fragment at *m/z* 179 (loss of hexoside moiety) which is characteristic of deprotonated caffeic acid. Additional fragments at *m/z* 161 and *m/z* 135 were also obtained, as reported by Grati et al., 2022 [47]. Ion x48 was characterized by *m/z* 205.1 which was found to be strongly co-eluting with *m/z* 331 and *m/z* 169 which was speculated to be a galloyl glucoside isomer [48]. Furthermore, ion x114 was characterized by *m/z* 357 at 9.6 min, with fragment ions *m/z* 135 and *m/z* 86, and was tentatively identified as a dimer of caffeic acid; however, MS/MS fragmentation was not conducted by the authors in the literature [49]. 

In the case of PAC and c-PAC%, the analyzed Chilean wines as well as the analyzed wines from Italy (Sicily/Puglia) were more characteristic of the prodelphinidin dimers (*m/z* 611). The analyzed wines from Argentina were separated mainly due to tetrameric prodelphinidins (*m/z* 1171). The percent ratios (%C-4) and (%C-5) were correlated with the analyzed wines from Italy (Sicily/Puglia), while the percent ratio (%C4-OH) was correlated with the analyzed Chilean wine. For these wines, the phenolic compounds that were positively correlated with the % ratios were ions x48 (*m/z* 205 at 2.9 min), x66 (*m/z* 111 at 5.2 min), x74 (*m/z* 375 at 5.9 min), and x151 (*m/z* 287 at 13.9 min). Ion x74 with *m/z* 375 at 5.9 min was tentatively assigned to a shikimic acid dimer or another acid with the same composition but condensed with acetaldehyde in an acetyl form. The result would exactly give a loss of shikimic acid, a peak of shikimic acid, as shown in the spectrum also reported in the Supplementary File S2 (*m/z* 174), and the monomeric shikimic (or another isomeric species and the acetylated form of shikimic acid). Ion x151 could be tentatively identified as limocitrin with its precursor ion *m/z* 346 and partially similar MS^2^ spectra of *m/z* 241, *m/z* 215, *m/z* 179, *m/z* 135, and *m/z* 109 [50]. 

### 3.4. Regression Models 

#### 3.4.1. Partial Least-Squares Regression (PLS-R) for the OQJ Score of Pinot Gris Wines

**Figure 7:**
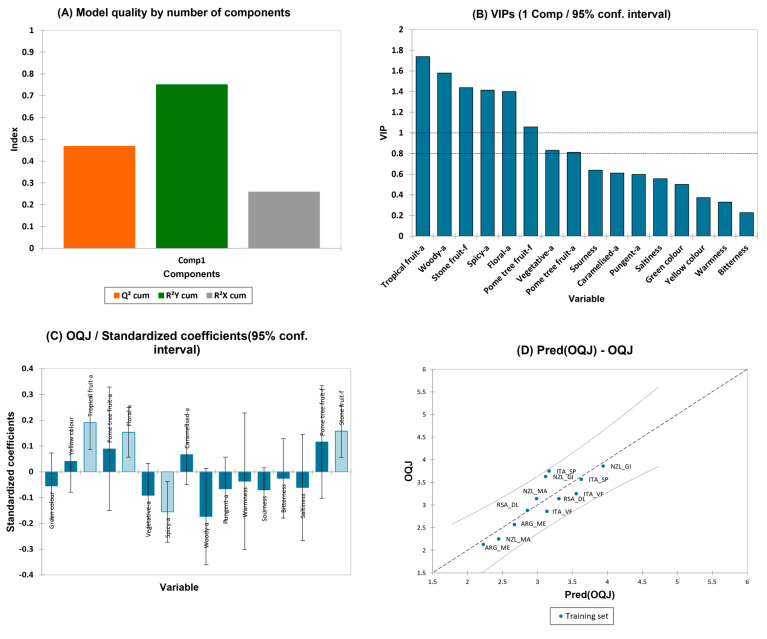
PLS data for the overall quality judgement over the sensory attributes of Pinot Gris wines. (**A**) Histogram of model quality by the number of components with Q^2^, R^2^Y, R^2^X indexes; (**B**) VIP for the sensory variables; (**C**) effects of the variables on the PLS equation and (**D**) PLS-predicted vs. experimental values of overall quality judgement.

To study which combination of sensory variables had greater impact on the overall quality judgment (OQJ) of Pinot Gris wines, a PLS regression method was used. The PLS model was built with overall quality as the Y-vector (dependent variable) and the sensory attributes as the X-matrix (independent variables, regressors). The PLS model for OQJ considering all the sensory descriptors as regressors built on one component showed the best performance, with a Q^2^ (cum) of 0.5, a R^2^X(cum) of 0.26, and a R^2^Y(cum) of 0.75 (Figure 7A). The VIP graph in (Figure 7B) allows us to visualize the most important variables influencing the model of the overall quality (VIP > 0.8). The most important variables were “tropical fruit” aroma, “woody” aroma,” floral” aroma, “stone fruit” flavor, “spicy” aroma, and “pome tree fruit” flavor. The variables “yellow” color, “tropical fruit” aroma, “pome tree fruit” aroma, “floral” aroma, “caramelized” aroma, “pome tree fruit” flavor, and “stone fruit” flavor showed a positive contribution in the regression model, while the variables “green” color, “vegetative” aroma, “spicy” aroma, “woody” aroma, “pungent” aroma, “warmness”, “sourness”, “bitterness”, and “saltiness” showed negative effect (Figure 7C). Figure 7D shows the experimental observations against the model predictions of the overall quality judgement. There were no observations seen outside the confidence intervals (α = 95%). The full regression model is reported in the Supplementary File S3—PG OQJ PLS.

#### 3.4.2. Partial Least-Squares Regression (PLS-R) for the Olfactory Attributes of Pinot Gris Wines

**Figure 8:**
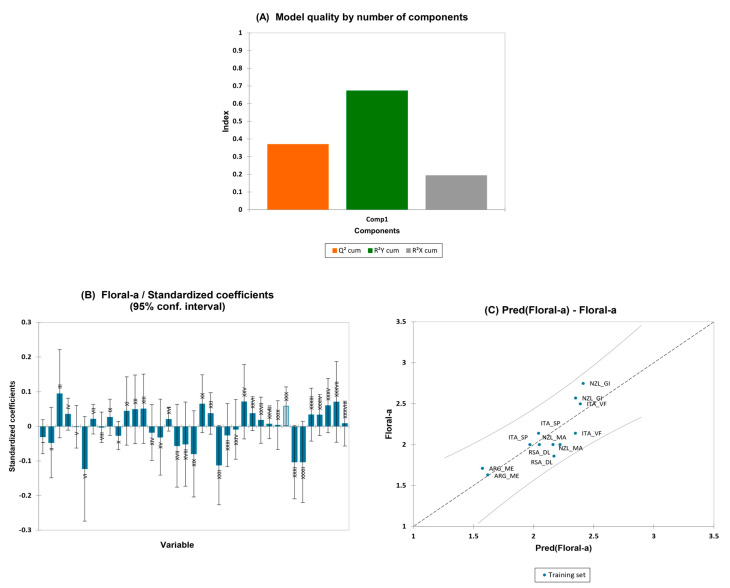
(**A**–**C**) PLS 1- histogram of model quality, Q^2^, R^2^Y, R^2^X indexes; effects of variables on the PLS equation and predicted vs. experimental observations for the olfactory attributes in Pinot Gris wines’ floral aroma.

Again, the PLS regression model was built with an individual VIP sensory descriptor from the previous paragraphs as the Y-matrix, and the volatile compounds as the X-matrix. “Woody”, “spicy”, and “tropical fruit” aroma attributes were tested in the PLS regression but did not show a good quality and, therefore, were not included in the manuscript. However, “floral” aroma was the only variable that showed predictive relevance. The PLS model of “floral” aroma had a Q^2^ of 0.4, R^2^Y (cum) of 0.7 and R^2^X (cum) of 0.2 considering all the variables (Figure 8A). The VIPs graph allowed us to observe which variables had the most influence on the “floral” aroma, as reported in Supplementary File S1—Table S18. The most important volatile compounds for this model were “VI: isopentanol”, “XXII: ethyl succinate”, “XXXI: ethyl isopentyl succinate”, “XXXII: phenylethyl alcohol”,” III: isobutyl alcohol”, “XIX: ethyl 2-furoate”, “XXV: ethyl phenylacetate”, “XXXVII: decanoic acid”, “XX: ethyl decanoate”, “XXXV: ethyl hexadecanoate”, and “XXX: isoamyl decanoate”. Figure 8C shows the regression graph of the predicted olfactory variables vs. the experimentally measured olfactory values for “floral” aroma. No data were found outside the confidence intervals (α = 95%). The results of the regression model are presented in Supplementary File S3—PG FLORAL PLS.

#### 3.4.3. Partial Least-Squares Regression (PLS-R) for the OQJ Score of Pinot Noir Wines

There was no significant correlation of the overall quality with any of the sensory descriptors found from ANOVA (Pearson’s correlation, *p*-value < 0.05) for Pinot Noir wines. Consequently, no tested regression model could provide an acceptable quality for describing the data, showing that a combination of variables that could give a significant model could not be found. There was no specific trend between the different samples and the overall quality itself, except when some outliers were included in the model training set. Univariate statistical analysis also showed no significant correlation between OQJ and specific sensory parameters. Many trials were conducted with different techniques, but the Q^2^ observed was very low. The result of the regression model is presented in Supplementary File S3—PN OQJ PLS.

#### 3.4.4. Partial Least-Squares Regression (PLS-R) for the Olfactory Attributes of Pinot Noir Wines

Nonetheless, for Pinot Noir wines, a PLS1 regression model was built by taking each sensory olfactory variables separately as the dependent Y-matrix and the volatile compounds as the independent X-matrix. The most correlating volatile compounds with the sensory olfactory attributes were considered for this model. Only the variables that showed predictive relevance are shown here, which were “green bell pepper” aroma, “cherry” aroma, “licorice” aroma, and “fresh wood” aroma. 

The PLS with two factors for “green bell pepper” aroma had a Q2 (cum) of 0.4, a R2X(cum) of 0.6, and a R2Y(cum) of 0.8. The model for “cherry” aroma had a Q2 (cum) of 0.2, a R2X(cum) of 0.6, and a R2Y(cum) of 0.7, which was also built with two components. The PLS model built with two factors for “licorice” aroma had a Q2 (cum) of 0.2, a R2X(cum) of 0.7, and a R2Y(cum) of 0.6. Lastly, for “fresh wood” aroma, the model was built with three factors and had a Q2 (cum) of 0.4, a R2X(cum) of 0.8, and a R2Y(cum) of 0.8.

**Figure 9:**
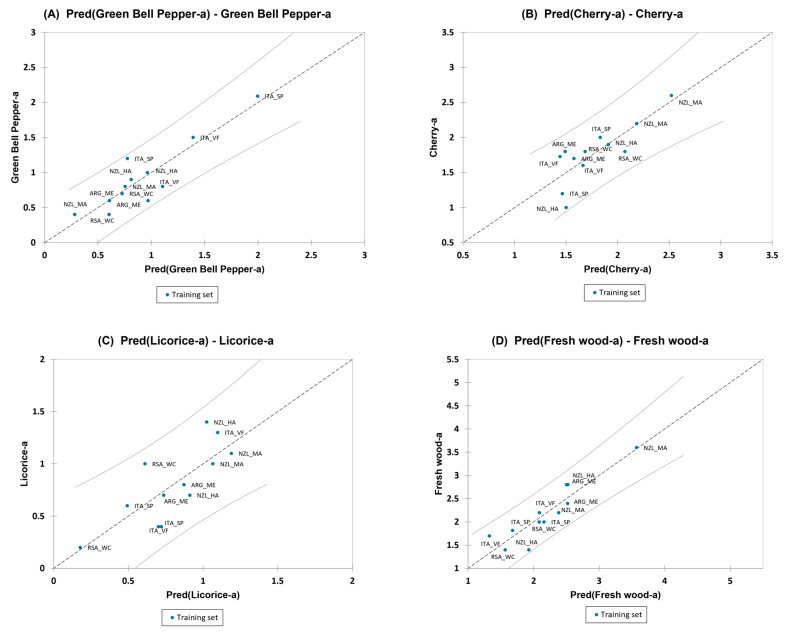
(**A**–**D**) The regression graphs for predicted vs. experimental data of “green-bell pepper” aroma, “cherry” aroma, “licorice” aroma, and “fresh wood” aroma of Pinot Noir wines.

The VIP graph of the PLS1 model for each individual sensory attribute is shown in Supplementary File S1—Tables S19–S22. Figure 9A–D shows the regression graph of the predicted vs. the experimental data for “green bell pepper” aroma, “cherry” aroma, “licorice” aroma, and “fresh wood” aroma, respectively. The R2, standard deviation, RMSE, and MSE are reported for the above-mentioned olfactory variables used in the regression in the following Table 6.

For “green bell pepper” aroma, the compounds “III: isopentanol”, “VIII: 1-hexanol”, “XVI: benzaldehyde”, “XXXIII: ethyl phenylacetate”, and “XLIII: ethyl isopentyl succinate” showed a positive influence in the regression model, while the compounds “VI: furfuryl ethyl ether ”, “XI: acetic acid”, “XIV: ethyl sorbate”, “XXVIII: ethyl 9-decenoate”, “XLII: trans-whiskey lactone ”, “XLIV: 4-ethylguaiacol”, and “XLVII: 4-ethylphenol” showed a negative effect. For “cherry” aroma, the compounds that showed a positive contribution in the model were “VI: furfuryl ethyl ether”, “VIII: 1-hexanol”, “XVI: benzaldehyde”, “XXVIII: ethyl 9-decenoate”, and “XLVII: 4-ethylphenol”, while the compounds “III: isopentanol” and “XXI: isoamyl lactate” showed a negative effect. For “licorice” aroma, the compounds “X: furfural”, “XVI: benzaldehyde”, “XXI: isoamyl lactate”, “XXX: 4-tert-butylcyclohexanol”, “XL: hexanoic acid”, “XLI: benzyl alcohol”, and “XLII: trans-whiskey lactone” showed a positive effect, while the compounds with a negative influence were “III: isopentanol”, “VI: furfuryl ethyl ether”, “XIV: ethyl sorbate”, “XLIII: ethyl isopentyl succinate”, “XLIV: 4-ethylguaiacol”, “XLVII: 4-ethylphenol”, and “XXIV: ethyl 2-furoate” Lastly, for “fresh wood” aroma, very few volatile compounds had a positive influence on the model, but included the compounds “XXVIII: ethyl 9-decenoate”, “XXIV: ethyl 2-furoate”, and “XXXVIII: β-damascenone”, while compounds “VIII: 1-hexanol”, “XIV: ethyl sorbate”, “XXX: 4-tert-butylcyclohexanol”, “XL: hexanoic acid”, “XLIII: ethyl isopentyl succinate,” and “XLVII: 4-ethylphenol” had a negative effect. 

The full regression model is presented in Supplementary File S3—PN GBP PLS, PN CH PLS, PN LI PLS, and PN FW PLS, respectively. 

### 3.5. Radar Plots 

A qualitative comparison between the overall quality of the wines obtained from the assessment by two different panels was performed using a spider plot. The term for quality rating by the German panel was reported as overall wine sensory score “OWS” (ranging from 0 to 5) and can be comparable to the score reported by our panel as overall quality judgement, or “OQJ”, ranging from 0 (lowest quality) to 5 (highest quality). 

**Figure 10:**
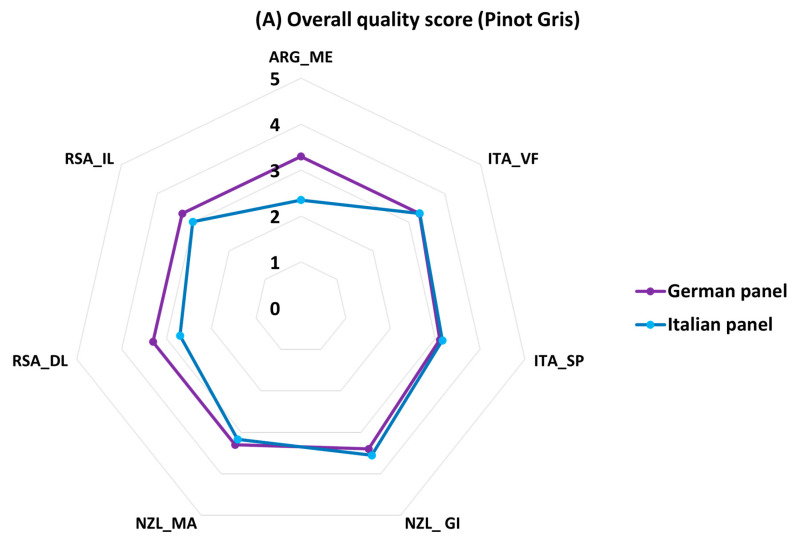

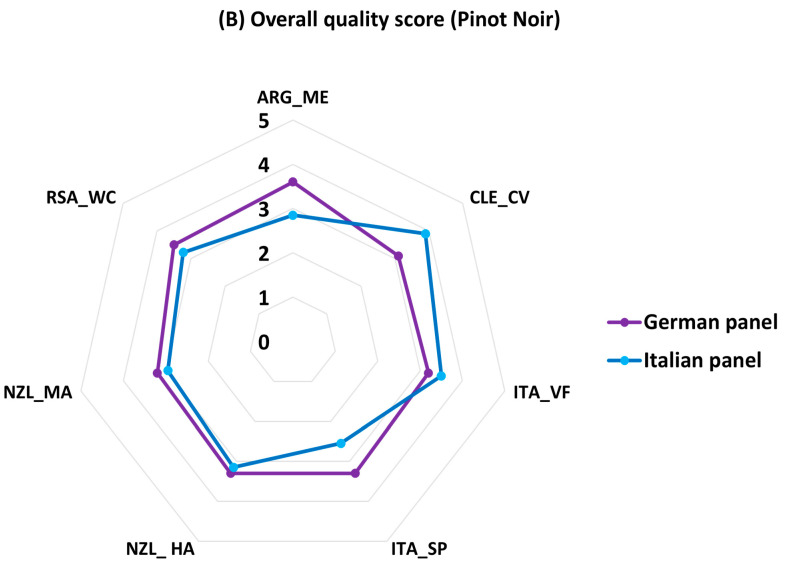
(**A**) Spider plot of the overall quality score (average values of each panel) given by the German and the Italian panels for Pinot Gris. (**B**) Spider plot of the overall quality score (average values of each panel) given by the German and the Italian panels for Pinot Noir.

For Pinot Gris wines (Figure 10A), both the German and the Italian panels gave higher scores to the analyzed wines from New Zealand (Gisborne), which were characteristic of floral, fruity, and tropical notes and yellow color for the Italian panel, while the German panel described them as also slightly fruity, aromatic, green-yellow and acidic. The lowest score on the other hand was given to the analyzed Argentinian wines by the Italian panel, with attributes, such as pungent, vegetative, and sour, while the German panel gave the lowest score to the analyzed wine from Italy (Sicily/Puglia), which was described as more ripe, less aromatic, and discreetly fruity. 

An obvious disparity in the choices between the two panels can be observed in Figure 10B for Pinot Noir. The Italian panel gave the highest score to the wine styles from Chile (Central Valley) which had more spicy, red fruit and woody notes. On the contrary, the German panel gave the highest score to the Pinot Noir wine styles from Argentina (Mendoza), which had light, subtle woody, and red fruit notes. The Italian panels gave the lowest overall quality scores to the analyzed wines from Italy (Sicily/Puglia). These wines were more characteristic of woody and spicy notes with sweet and astringent taste. Conversely, the German panel gave the lowest score to the wine styles from Chile, which had tannic, subtle woody, and spicy notes and were slightly oxidized.

## 4. Discussion

This study characterized two monovarietal wines Pinot Gris and Pinot Noir and defined relations between the sensory attributes and the measured sensory quality which were unique for both varieties. The term quality is hard to define due to the multidimensional differences featured by the product which comprises a wide range of both intrinsic and extrinsic factors; here, it has been described as an objective assessment of all the sensory features [5,51].

Previous studies have reported attributes, such as “tropical/pineapple”, “lemon/lime”, “bruised apple”, “perfume/floral”, “capsicum”, “grassy”, “dried fruit aroma”, “honey suckle”,” stone fruit”, “pome”, “rose”, “melon”, “ginger”, “grapefruit”, “lilac”, and “lavender” [52,53], as sensory descriptors for Pinot Gris wines. Most of these descriptors were also true for the analyzed commercial wines in this study. However, more detailed lexicons (differentiated by color, olfactory, and gustatory attributes) have been reported in this article, and the results from a one-way ANOVA showed that “green” color, “yellow” color, and “floral” aroma were significant attributes for Pinot Gris wines across different international origins and, thus, different styles. For Pinot Noir wines, sensory descriptors, such as “fresh berry (strawberry, raspberry, black currant)”, “(berry jam-strawberry, raspberry, blackberry)”, “cherry”, “prune”, “spicy (black pepper, cloves)”, “mint/eucalyptus”, “earthy (potato, mushroom)”, “leather”, “vegetal (green bean, green tea)”,“smoke/tar”, “berry by mouth”, “bitterness”, “astringency”, “tart fruit aroma”, “sweet fruit aroma”, “sweet aroma”, “pepper spice aroma”, “baking spice aroma”, “tobacco aroma”, and “acidity” [54,55], have been reported in the literature. Most of them were also true for the wines in this study. Moreover, out of the lexicons developed by the panel, the most significant sensory attributes for Pinot Noir wines across the different international origins (and, therefore, different styles) were “violet-red” color, “red-brown” color, “licorice” aroma, and “warmness”.

As with the previous approach described by Poggesi et al., 2022 [6] on Chardonnay and Sauvignon Blanc wines, MFA helped visualize correlations among different wine samples of Pinot Gris and Pinot Noir and different chemical and sensory variables with respect to overall quality score given by an Italian panel. For Pinot Gris, the results showed that the analyzed wines from New Zealand (Gisborne) were characterized by attributes, such as “floral” aroma, “stone-fruit” flavor, “yellow” color, “caramelized” aroma, and “tropical fruit” aroma that correlated with the OQJ. The analyzed wines from Argentina, on the other hand, were negatively correlated with the OQJ given by the Italian panel, with characteristics, such as “green” color, “woody” aroma, and “warmness”. Additionally, the volatile compounds that correlated with the OQJ were isobutyl alcohol, benzenebutanal, ethyl phenylacetate, and phenethyl acetate. Tentatively identified phenolic compounds that showed a correlation with the OQJ were adducts of sulfonated caftaric acid, dihydroxybenzoic acid hexoside, and astilbin. For Pinot Noir, the OQJ was positively correlated with sensory attributes, such as “licorice” aroma, “cloves” aroma, “fresh wood” aroma, “red fruit” flavor, “cherry” aroma, and “spicy” flavor, which characterized the analyzed wines from Chile. Also, for the Pinot Noir variety, the analyzed wines from Argentina correlated negatively with the OQJ, with sensory attributes, such as “sourness”, “rose” aroma and “spicy” flavor. The volatile compounds that correlated with the OQJ were isoamyl acetate, ethyl hexanol, ethyl sorbate, phenethyl acetate, and phenol,4-ethyl. The analyzed Chilean wines were also characterized by anthocyanin malvidin-3-O-(6′-acetyl)-glucoside), which correlated with OQJ. 

In the case of proanthocyanidins for both wines, different non-cyclic and cyclic PAC differentiated the origins, but not clearly. This result was also found in previous work published by Poggesi et al., 2022 [6], where only some differences due to the origins were found according to this variable. Instead, c-PAC variables and the related %-ratio indexes featured in that study were more prominent as varietal markers, an aspect not investigated in this study. However, interestingly for both Pinot Gris and Pinot Noir, the %-ratio chemical indexes %C-4 and %C-5 separated one origin, which was Italy (Sicily/Puglia), from all the rest of the samples. The correlations between these indexes and the phenolic compounds were also observed. The conditions that favored the accumulation of c-PAC possibly also favored the higher abundance of the phenolic compounds present in these samples. 

Furthermore, regression models have been reported, in which the relationship between the overall quality score (OQJ) and sensory attributes are shown, for both varieties. In Pinot Gris wines, the descriptors with large effect on the regression model (VIP > 1) were “yellow” color, “tropical fruit” aroma, “floral” aroma, “stone fruit” flavor, “caramelized” aroma, and “pome tree fruit” flavor, while for Pinot Noir, unfortunately, there was no significant model that could describe which sensory attributes contributed to the overall quality of the wines. Various other regression models were also tested, and the model with the best results has been reported, with the focus being solely on the relation between the volatile compounds and the sensory aroma attributes. However, a PLS-1 regression was performed again with the volatile compounds as explanatory variables and each sensory olfactory variable as the dependent variable for both varieties. Identification of the volatile compounds in both Pinot Gris [56,57] and Pinot Noir [58,59,60] wines have been reported by many authors; however, little information regarding the relationship between the volatile compounds and the sensory attributes is available. The results from this study showed that in Pinot Gris wines, for the “floral “aroma, the volatile compounds isobutyl alcohol, linalool, ethyl phenylacetate, and decanoic acid showed a positive effect. Indeed, previous studies have shown that volatile compounds, such as hexanoic acid, octanoic acid, linalool, and decanoic acid (volatile fatty acids), were identified in Pinot Gris wines, which were responsible for imparting a floral aroma [29,53]. For Pinot Noir, volatile compounds, such as isopentanol, 1-hexanol, benzaldehyde, ethyl phenylacetate, and ethyl isopentyl succinate, showed a positive influence on the “green bell pepper” aroma. Indeed, 1-hexonal has been reported in many studies as a compound with a strong positive influence on imparting vegetal notes in wines [61,62,63]. For “cherry” aroma, the compounds were furfuryl ethyl ether, 1-hexanol, benzaldehyde, ethyl 9-decenoate, and also 4-ethylphenol. The “licorice” aroma was positively influenced by a combination of volatile compounds, namely furfural, benzaldehyde, isoamyl lactate, 4-tert-butylcyclohexanol, hexanoic acid, benzyl alcohol, and trans-whiskey lactone. Very few compounds appeared to have a positive effect on the “fresh wood aroma” but included ethyl 9-decenoate, ethyl 2- furoate, and β-damascenone. Along with this investigation, there are studies where the relation between flavor and aroma with the volatile compounds, and the relation between visual and gustatory with the non-volatile phenolic compounds, have been studied, which could provide information regarding the interplay of these analyzed compounds with the sensory variables [6].

Although the identity of single-varietal Pinot Gris and Pinot Noir wines from different countries has been studied in this work, it should be emphasized that the number of samples is far from representative of a particular country’s wine style. Nonetheless, the overall quality scores given by an Italian and a German panel were qualitatively compared to understand the relative overall quality judgment given by the two panels and offer an indication of how the trader companies or wineries could strategize marketing these wines globally [64,65,66]. These cited studies also evidenced significant correlations between the quality scores given by French and Spanish panels of experts and the evaluations by the consumers of same nationality, highlighting an effect from the panels’ intrinsic composition factors.

As mentioned by Poggesi et al., 2022, it is important to take into consideration the composition of the panel and their origin and to conduct interlaboratory tests with standardized methods for a thorough conclusion to elucidate the acceptance behavior of consumers worldwide. That being the case, the German panel in this study were accredited ones, while the Italian panels were formed from staff and students familiar with wine tasting and underwent training for these specific wines. For this reason, only the quality parameter was compared between the two and not the specific attributes. The results from this study showed that panels from both countries gave higher scores to the analyzed Pinot Gris wines from New Zealand (Gisborne) that were characteristic of fruity and aromatic notes, but it was clear that the German panel gave a higher OQJ to Pinot Gris for its acidity. For Pinot Noir, the Italian panel gave the highest score to the wine styles from Chile (Central Valley), characteristic of more spicy, red fruit flavor and woody notes, while the German panel gave a higher score to the wine styles from Argentina (Mendoza), characterized by woody and red fruit flavors as sensory attributes. 

In conclusion, commercial quality, being a subjective and multifaceted term for wine, in this study has been linked with sensory and chemical measurements as also reported by Hopfer, 2015 [67]. These produced results can not only be useful for further research and validations with an exhaustive set of samples with regards to the origin and panel composition, but also serve as a tool beneficial for addressing the commercial strategy of the winery or trader companies for selling their products internationally.

## Figures and Tables

**Table 1:** Code, origin, and basic oenological parameters of Pinot Gris samples. The alcohol content (%vol), residual sugars (RS, g/L) and titratable acidity (TA, g/L tartaric acid) were analyzed by official methods according to OIV [10].

Code	Origin of Wines	Country of Origin	Vintage	Alcohol (% Vol)	RS (g/L)	TA (g/L Tartaric Acid)	pH	Time and Temperature of Fermentation
PG_NZL-MA	Marlborough (South Island)	New Zealand	2020	13.0	3.7	5.7	3.3	14 to 21 days at 15 °C
PG_NZL_GI	Gisborne/East Coast (North Island)	New Zealand	2020	13.0	5.9	5.2	3.4	14 days at 12–14 °C
PG_ARG_ME	Mendoza	Argentina	2020	14.0	2.0	5.6	3.4	12 days at 13 °C
PG_RSA_DL	Western Cape (Dry land)	South Africa	2020	12.5	3.4	5.3	3.3	13 °C
PG_RSA_IL	Western Cape (Irrigated area)	South Africa	2020	13.0	1.6	5.4	4.0	n/a
PG_ITA_SP	Sicily/Puglia	Southern Italy	2020	12.0	3.2	5.4	3.3	8 days at 14–16 °C
PG_ITA_VF	Veneto/Friuli	Northern Italy	2020	12.7	2.9	6.4	3.3	8–10 days at 18 to 20 °C

**Table 2:** Code, origin, and basic oenological parameters of Pinot Noir samples. The alcohol content (%vol), residual sugars (RS, g/L), and titratable acidity (TA, g/L tartaric acid) were analyzed by official methods according to OIV [10].

Code	Origin of Wines	Country of Origin	Vintage	Alcohol (% Vol)	RS (g/L)	TA (g/L Tartaric Acid)	pH	Time and Temperature of Fermentation
PN_NZL_MA	Marlborough (South Island)	New Zealand	2020	13.5	1.5	4.9	3.7	7 days at 30 °C
PN_NZL_HA	Hawkes Bay (North Island)	New Zealand	2019	13.0	2.1	6.23	3.6	10–14 days at 20–25 °C
PN_ARG_ME	Mendoza	Argentina	2020	15.4	3.2	7.5	3.6	8 days at 25 °C
PN_RSA_WC	Western Cape (Dry land)	South Africa	2020	13.5	2.3	5.7	3.4	7 days at 23 °C
PN_CLE_CV	Central Valley	Chile	2020	12.7	2.8	5.5	3.4	6 days at 25–28 °C
PN_ITA_SP	Sicily/Puglia	Southern Italy	2020	12.7	6.5	6.0	3.4	15 days at 18–20 °C
PN_ITA_VF	Veneto/Friuli	Northern Italy	2020	13.3	0.9	4.8	3.5	8–10 days at 28 °C

**Table 3:** Climate details for the different origins of the samples.

Origins	Zone	Climate Description	Temperature Range (Yearly/°C)	Rainfall (Yearly/mm)	Climate Classification
Marlborough	South Island, New Zealand	Warm and temperate. Heavy rainfall even in driest months. Oceanic climate [11]	12.0 °C (53.7 °F)	874 mm (34.4 inches)	Cfb
Gisborne	North Island, East Coast, New Zealand	Oceanic climate. Warm and temperate. Significant rainfall throughout the year. Even the driest month still has a lot of rainfall. Climate is considered to be Cfb according to the Köppen–Geiger classification [12]	13.9 °C (56.9 °F)	929 mm (36.6 inches)	Cfb
Hawkes Bay	North Island, New Zealand	Mild, generally warm, and temperate with a significant amount of rainfall during the year [13]	7.2–19.5 °C (45–56.9 °F)	59–110 mm (2–4 inches)	Cfb
Mendoza	Argentina	Hot and clear summer, cold and cloudy winter, and dry all year round [14]	16.3 °C (61.4 °F)	492 mm (19.4 inches)	Cfb
Central Valley	Chile	Mediterranean climate with cool, dry summers. Mild rainy winters [15]	10–12 °C (50–53.6 °F)	72–99 mm (2.8–3.9 inches)	Csb
Western Cape (dry land)	South Africa	Dry, summer rainfall, relatively warm in winter [16]	Below 30 °C	464 mm (18 inches)	BWk
Western Cape (irrigated area)	South Africa	Mediterranean with warm and dry summers. Mild and moist winters. Low summer rainfall [16]	Below 30 °C	464 mm (18 inches)	BWk
Sicily/Puglia	Southern Italy	Mediterranean with hot summers and short mild winters [17] Mediterranean climate with hot sunny summers and mild winters, dry [18]	18.25 °C (64.85 °F)/9–24 °C (48–75 °F)	55.03 mm (2.17 inches)/99.3 mm (4 inches)	Csa/Csa
Veneto/Friuli	Northern Italy	Moderately continental hill and plain areas; alpine region characterized by cool summers and cold winters with frequent snowfalls [19] The climate in Friuli Venezia Giulia ranges from the sub-Mediterranean climate of the coastal areas to the more humid temperate climate of the plains, to the Alpine climate in the mountains [20]	15.93 °C (60.67 °F)/14.93 °C (58.87 °F)	152.54 mm (6.01 inches)/163.49 mm (6.44 inches)	Cfa/Cfb

Cfb = Temperate oceanic climate; Csb = warm summer Mediterranean climate; BWk = cold desert climate; Csa = hot summer Mediterranean climate; Cfa = humid subtropical climate.

**Table 4:** Visual, aromatic, and gustatory attributes for Pinot Gris. The abbreviations ‘a’ refers to aroma and ‘f’ refers to flavor.

Wine	Attributes	Descriptors	Description
PINOT GRIS	VISUAL		
	GREENISH		From pale grass to an intense green
	YELLOWISH		From pale straw to a rich yellow
	OLFACTORY		
	Pome tree fruit-a	Green apple, pear	Tree fruit (green apple, pear)
	Caramelized-a	Chestnut honey	Chestnut–honey
	Woody-a	Oak wood	Fresh or burnt wood
	Pungent-a	Alcohol	Strong sensation from alcohol
	Tropical fruit-a	Banana, pineapple	Tropical fruit (banana, pineapple)
	Vegetative-a	Green bell pepper	Cut green bell pepper
	Spicy-a	Clove, white pepper	Cloves and white pepper
	Floral-a	Rose, Jasmine green tea	Rose water and fresh scent of wildflowers, jasmine, green tea (infused)
	GUSTATORY		
	Warmness	Alcohol	Burning sensation in the mouth
	Sourness	Acid (tartaric, malic, lactic acid)	Acid taste resembling vinegar, ripe apple
	Bitterness	Caffeine	Bitter taste typical for coffee beverage
	Saltiness	Sodium chloride	Salty taste typical for salt
	FLAVOR		
	Pome tree fruit-f	Apple/pear	Typical of pome tree fruits (apple, pear)
	Stone fruit-f	Apricot/peach	Typical of stone fruits (apricot, peach)
*OVERALL QUALITY JUDGEMENT (OQJ)*		A score given by the panel on the sensory quality of the product	

**Table 5:** Visual, aromatic, and gustatory attributes for Pinot Noir. The abbreviations ‘a’ refers to aroma and ‘f’ refers to flavor.

Wine	Attributes	Descriptors	Description
PINOT NOIR	VISUAL		
	Violet-red		From violet to ruby red
	Red-brown		From red to brown
	OLFACTORY		
	Woody-a	Fresh wood	Fresh oak wood (woody)
		Coffee	Coffee
	Spicy-a	Cloves	Cloves
		Black pepper	Black pepper
		Licorice	Licorice
	Red fruit-a	Cherry	Cherries
		Strawberry	Strawberries
	Dried fruit-a	Strawberry jam	Strawberry jam (cooked)
	Floral-a	Rose	Rose water
	Vegetative-a	Green bell pepper	Fresh-cut green bell pepper
	GUSTATORY		
	Bitterness	Caffeine	Bitter taste typical from coffee beverage
	Astringency	Alum	Astringent somesthetic sensation (tannins)
	Sweetness	Sweetness (glucose, fructose, and sucrose)	Sweet taste in the mouth
	Warmness	Alcohol	Burning sensation in the mouth
	Sourness	Acid (tartaric, malic, lactic acid)	Acid taste resembling vinegar, ripe apple
	FLAVOR		
	Woody-f	Fresh oak and toasted tannins	Typical from toasted barrique
	Red fruit-f	Cherry and strawberry	Red fruits (strawberry and cherry)
	Vegetative-f	Green bell pepper	Fresh vegetable (green bell pepper)
	Spicy-f	Cloves	Cloves
		Nutmeg	Typical of nutmeg
		Licorice	Typical of licorice, anise
*OVERALL QUALITY JUDGEMENT (OQJ)*		A score given by the panel on the sensory quality of the product	

**Table 6:** Quality index for PLS regression on olfactory variables for Pinot Noir wines.

	Green Bell Pepper	Cherry	Licorice	Fresh Wood
R^2^	0.810	0.664	0.603	0.838
Std. deviation	0.235	0.266	0.259	0.302
RMSE	0.202	0.231	0.224	0.246
MSE	0.041	0.053	0.050	0.061

## Data Availability

Acquired data are available upon request.

## Supplementary Materials

The following supporting information can be downloaded at: https://www.mdpi.com/article/10.3390/foods13010018/s1, Supplementary File S1: Table S1: Results for 1st Training Session (Aroma) for Pinot Gris; Table S2: Results for 2nd Training Session (Aroma) for Pinot Gris; Table S3: Results for 2nd Training Session (Taste) for Pinot Gris; Table S4: Results for 2nd Training Session (Flavor) for Pinot Gris; Table S5: Results for 1st Training Session (Aroma) for Pinot Noir; Table S6: Results for 2nd Training Session (Aroma) for Pinot Noir; Table S7: Results for 2nd Training Session (Taste) for Pinot Noir; Table S8: Results for 3rd Training Session (Aroma)for Pinot Noir; Table S9: Results for 3rd Training Session (Taste )for Pinot Noir; Table S10: Results for 3rd Training Session (Flavor)for Pinot Noir; Table S11: List of identified volatile compounds in Pinot Gris; Table S12: List of identified volatile compounds in Pinot Noir; Table S13: List of tentatively identified phenolic compounds in Pinot Gris (PG) and Pinot Noir (PN) wines based on retention time. The compounds followed by an asterisk were assigned by observing the mass fragmentation, whereas all the other compounds were identified by injecting the related standard compounds. The data of the fragmentation are reported in Tables S20 and S21 in the Excel Supporting Information File S2; Table S14: Single ion monitoring (SIM) acquisition parameters for analysis of PAC in Pinot Gris and Pinot Noir wines, total scan cycle time = 500 ms; Table S15: Filtered PAC features observed in the LC-MS analysis for Pinot Gris; Table S16: Filtered PAC features observed in the LC-MS analysis for Pinot Noir; Table S17: Target identification of anthocyanins in Pinot Noir based on molecular ion [M-H]—(*m/z*) and lambda max (UV spectrum absorbance); Table S18: The variables’ importance in projection (VIP) in the PLS regression olfactory attribute “floral” aroma vs. volatile compounds for Pinot Gris; Table S19: The variables’ importance in projection (VIP) in the PLS regression olfactory attribute “green bell pepper” aroma vs. volatile compounds for Pinot Noir; Table S20: The variables’ importance in projection (VIP) in the PLS regression olfactory attribute “cherry” aroma vs. volatile compounds for Pinot Noir, Table S21: The variables’ importance in projection (VIP) in the PLS regression olfactory attribute “licorice” aroma vs. volatile compounds for Pinot Noir; Table S22: The variables’ importance in projection (VIP) in the PLS regression olfactory attribute “fresh wood” aroma vs. volatile compounds for Pinot Noir; Figure S1: Example of a targeted analysis for peonidin-3-glucoside. DAD absorbance spectrum; EIC (extracted ion chromatogram) targeting 463 *m/z* (precursor ion) and 303 *m/z* (product ion) at different retention times and MS2 fragments from ESI, reported as *m/z* ratio values, respectively; Supplementary File S2—Table S20 Pinot Gris and Table S21 Pinot Noir; LC-MS-MS spectra. Supplementary File S3—regression models.
