# Supplementary material for: Decoding the Identity of Pinot Gris and Pinot Noir Wines: A Comprehensive Chemometric Fusion of Sensory (from Dual Panel) and Chemical Analysis
Source: Foods. 2023 Dec 20;13(1):18. doi: 10.3390/foods13010018 (PMC10778262; doi:10.3390/foods13010018)
Supplement: Supplementary file 1 [file foods-13-00018-s001.zip › Supplementary file S1_Darnal et al.pdf]

Decoding the identity of Pinot Gris and Pinot Noir wines: A comprehensive chemometric fusion of sensory (from dual panel) and chemical analysis

Aakriti Darnal,[1,2] Simone Poggesi,[1,2,3] Edoardo Longo,[1,2]* Annagrazia Arbore,[1,2] and Emanuele Boselli[1,2]

AFFILIATIONS

[1] Oenolab, NOI Techpark Alto Adige/Südtirol, Via A. Volta 13B, 39100 Bolzano, Italy

[2] Free University of Bozen-Bolzano, Faculty of Agricultural, Environmental, and Food Sciences, Piazza Università 1, 39100 Bolzano, Italy

[3] Food Experience and Sensory Testing (Feast) Lab, Massey University, Palmerston North 4410, New Zealand

*corresponding author: edoardo.longo@unibz.it

Supplementary File

Table S1: Results for 1st Training Session (Aroma) for Pinot Gris

ATTRIBUTES	% CORRECT ANSWERS BLIND TEST	% CORRECT ANSWERS DESCRIPTOR TEST
GREEN TEA	20%	44%
ROSE	30%	56%
APPLE/PEAR MIX	100%	100%
GREEN BELL PEPPER	80%	100%
HONEY	50%	100%
OAK WOOD	70%	78%
CLOVE	70%	100%
WHITE PEPPER	70%	100%
ALCOHOL	65%	100%
PINEAPPLE	90%	100%
BANANA	35%	56%
**TOTAL**	**68%**	**73%**

Table S2: Results for 2nd Training Session (Aroma) for Pinot Gris

ATTRIBUTES	% CORRECT ANSWERS DESCRIPTOR TEST
GREEN TEA	50%
ROSE	40%
HONEY	90%
**TOTAL**	**60%**

Table S3: Results for 2nd Training Session (Taste) for Pinot Gris

ATTRIBUTES	% CORRECT ANSWERS TASTE TEST
WARMNESS	50%
BITTERNESS	60%
SOURNESS	90%
SALTINESS	100%
**TOTAL**	**60%**

Table S4: Results for 2nd Training Session (Flavour) for Pinot Gris

ATTRIBUTES	% CORRECT ANSWERS
APPLE/PEAR	60%
PEACH/APRICOT	45%
**TOTAL**	**53%**

Table S5: Results for 1st Training Session (Aroma) for Pinot Noir

ATTRIBUTES	% CORRECT ANSWERS BLIND TEST	% CORRECT ANSWERS DESCRIPTOR TEST
FRESH WOOD	55%	60%
COFFEE	100%	100%
CLOVES	50%	50%
BLACK PEPPER	70%	80%
LICORICE	90%	80%
CHERRY	40%	90%
STRAWBERRY	50%	40%
STRAWBERRY JAM	25%	90%
ROSE	30%	50%
GREEN BELL PEPPER	80%	100%
**TOTAL**	**59%**	**74%**

Table S6: Results for 2nd Training Session (Aroma) for Pinot Noir

ATTRIBUTES	% CORRECT ANSWERS DESCRIPTOR TEST
ROSE	78%
STRAWBERRY	22%
CHERRY	56%
CLOVES	100%
STRAWBERRY JAM	22%
FRESH WOOD	56%
**TOTAL**	**56%**

Table S7: Results for 2nd Training Session (Taste) for Pinot Noir

ATTRIBUTES	% CORRECT ANSWERS TASTE TEST
ASTRINGENCY	50%
BITTERNESS	60%
SOURNESS-TARTARIC	90%
SOURNESS-LACTIC	100%
**TOTAL**	**81%**

Table S8: Results for 3rd Training Session (Aroma)for Pinot Noir

ATTRIBUTES	% CORRECT ANSWERS DESCRIPTOR TEST
STRAWBERRY	67%
STRAWBERRY JAM	67%
**TOTAL**	**67%**

Table S9: Results for 3rd Training Session (Taste)for Pinot Noir

ATTRIBUTES	% CORRECT ANSWERS TASTE TEST
SWEETNESS (GLU-FRU-SUCR)	56%
WARMNESS	67%
ASTRINGENCY	89%
**TOTAL**	**70%**

Table S10: Results for 3rd Training Session (Flavour)for Pinot Noir

ATTRIBUTES	% CORRECT ANSWERS DESCRIPTOR TEST
WOODY	89%
RED FRUIT	100%
VEGETATIVE	100%
SPICY	67%
**TOTAL**	**89%**

Table S11: List of identified volatile compounds in Pinot Gris

S.N.	Compounds	Retention Times		Calculated Retention Index	Base mass (*m/z*)
		RT I (min)	RT II (sec)		
**I**	Ethyl acetate	5.2	0.7	949	43
**II**	Ethyl butanoate	8.7	1.4	1032	71
**III**	Isobutyl alcohol	11.7	0.6	1102	43
**IV**	Isoamyl acetate	12.5	1.6	1120	43
**V**	Ethyl hexanoate	17.5	1.7	1239	88
**VI**	Isopentanol	17.6	0.7	1241	55
**VII**	Hexyl acetate	19.0	1.7	1273	43
**VIII**	3-Ethyl-3-hexanol	19.3	0.9	1279	55
**IX**	Ethyl octanoate	26.3	1.7	1436	88
**X**	Acetic acid	27.2	0.9	1456	43
**XI**	Ethyl sorbate (isomer I)	28.1	1.3	1480	67
**XII**	Ethyl sorbate (isomer II)	29.4	1.3	1511	67
**XIII**	Benzaldehyde	29.5	1.0	1514	77
**XIV**	Nonanoic acid, 2-oxo-, methyl ester	29.8	1.2	1521	57
**XV**	Octanoic acid, phenyl ester	29.8	1.6	1521	57
**XVI**	Linalool	30.9	1.0	1549	41
**XVII**	2,3-Butanediol (I)	32.3	0.6	1583	45
**XVIII**	2,3-Butanediol (II)	33.1	0.6	1602	45
**XIX**	Ethyl 2-furoate	33.7	0.8	1616	95
**XX**	Ethyl decanoate	34.3	1.8	1633	88

**XXI**	Isopentyl octanoate	35.0	2.1	1648	70
**XXII**	Ethyl succinate	36.0	0.9	1668	101
**XXIII**	a-Terpineol	36.7	0.7	1683	59
**XXIV**	Vinyl decanoate	37.6	1.3	1702	43
**XXV**	ethyl phenylacetate	39.7	1.1	1746	91
**XXVI**	2-Phenethyl acetate	40.8	0.9	1769	104
**XXVII**	Benzenebutanal	40.8	2.0	1769	104
**XXVIII**	Ethyl dodecanoate	41.7	2.4	1789	88
**XXIX**	Hexanoic acid	42.1	0.9	1797	60
**XXX**	Isoamyl decanoate	42.3	1.9	1803	70
**XXXI**	Ethyl isopentyl succinate	44.0	1.1	1837	101
**XXXII**	Phenylethyl Alcohol	44.3	0.6	1850	91
**XXXIII**	Octanoic acid	49.2	0.5	2083	60
**XXXIV**	Sorbic Acid	51.9	0.9	2213	97
**XXXV**	Ethyl hexadecanoate	54.5	1.3	2336	88
**XXXVII**	Decanoic acid	55.0	0.5	2361	60
**XXXVIII**	2,4-Di-tert-butylphenol	55.6	0.6	2387	191

Table S12: List of identified volatile compounds in Pinot Noir

S.N.	Compounds	Retention Times		Calculated Retention Index	Base mass *(m/z)*
		RT I (min)	RT II (sec)		
**I**	Ethyl Acetate	4.7	0.8	937	43
**II**	Isoamyl acetate	12.1	1.7	1109	70
**III**	Isopentanol	16.6	0.6	1217	55
**IV**	Ethyl hexanoate	17.4	1.8	1232	88
**V**	Hexyl acetate	18.9	1.3	1217	56
**VI**	Furfuryl ethyl ether	19.6	1.1	1287	81
**VII**	Ethyl lactate	22.4	0.6	1349	75
**VIII**	1-Hexanol	22.9	0.6	1361	56
**IX**	Ethyl octanoate	26.2	1.8	1429	88
**X**	Furfural	27.2	0.8	1460	96
**XI**	Acetic acid	27.3	0.5	1455	43
**XIII**	2-Ethyl-1-hexanol	28.7	0.8	1493	57
**XIV**	Ethyl sorbate	29.1	1.0	1503	67
**XV**	2(1H)-Naphthalenone, 3,4,4a,5,6,7-hexahydro-1,1,4a-trimethyl-	29.5	2.2	1516	93
**XVI**	Benzaldehyde	29.6	0.9	1515	77
**XVII**	β-ionone	29.7	1.8	1522	41
**XX**	Linalyl acetate	30.9	0.7	1549	41
**XXI**	Isoamyl lactate	31.8	0.7	1570	45
**XXII**	2,3-Butanediol	32.4	0.6	1598	45

**XXIV**	Ethyl 2-furoate	33.6	0.7	1615	95
**XXV**	Ethyl decanoate	34.3	1.6	1630	88
**XXVII**	Ethyl succinate	36.0	1.0	1666	101
**XXVIII**	Ethyl 9-decenoate	36.2	1.4	1673	88
**XXIX**	α-Terpineol	36.7	0.9	1683	59
**XXX**	4-tert-Butylcyclohexanol	36.8	0.9	1688	57
**XXXII**	Z-9-Tetradecenyl acetate	39.3	1.2		55
**XXXIII**	ethyl phenylacetate	39.6	1.2	1745	43
**XXXIV**	trans-Cubebol	40.2	1.6		161
**XXXV**	2-Phenethyl acetate	40.7	1.2	1768	104
**XXXVI**	4-Methyl-6-phenyltetrahydro-1,3-oxazine-2-thione	40.7	1.9		104
**XXXVII**	Benzenebutanal	39.7	0.8	1746	104
**XXXVIII**	β -damascenone	40.9	1.2	1771	69
**XXXIX**	Cyclobuta[a]dibenzo[c,f]cycloheptadiene, 7-oxo-	40.7	2.2		104
**XL**	Hexanoic acid	42.0	0.8	1797	60
**XLI**	Benzyl alcohol	43.0	0.7	1817	79
**XLII**	(trans-whiskey lactone)	43.4	0.9	1824	99
**XLIII**	Ethyl isopentyl succinate	43.9	1.2	1836	101
**XLIV**	Phenol, 4-ethyl-2-methoxy-	47.9	1.0	2029	137
**XLV**	Nerolidol	48.4	1.0	2043	60
**XLVI**	Octanoic acid	49.1	1.2	2081	60
**XLVII**	Phenol, 4-ethyl-	52.5	0.5	2241	107

**XLVIII**	Hexadecanoic acid, ethyl ester	54.5	1.4	2335	88
**XLIX**	n-Decanoic acid	55.0	0.6	2361	60
**L**	2,4-Di-tert-butylphenol	55.5	0.6	2385	191

Table S13: List of tentatively identified phenolic compounds in Pinot Gris (PG) and Pinot Noir (PN) wines based on retention time. The compounds followed by asterisk were assigned by observing the mass fragmentation, whereas all the other compounds were identified by injecting the related standard compounds. The data of the fragmentation is reported in Table S20 and S21 in the Excel Supporting Information File S2.

Code PG	Code PN	Compound assignment	R.T. (min)	ESI- mass (m/z)	MS^2 fragments (*m/z*)
x23		Gallic acid	2.9	169	
x29		Sulfonated caftaric acid (tentative)*	4.3	391	259,241,213, 161, 149
x55	x84	Glycosylated derivative of dihydroxybenzoic acid*	6.6	315	153,123
x57		Kaempferol glucuronide (tentative)*	6.7	157	
	x57	β – glucopyranoside*	4.3	345	161,139,97
x61	x88 (6.9)	Caftaric acid	6.9	311	149,135
x75	x92 (7.1)	Glutathionyl caftaric acid, trans isomer (tentative)*	7.2	616	
x81		Cis-coutaric acid*	8.1	295	
	x101	Caffeic acid hexoside*	8.9	341	179, 161, 135, 71
x88	x104 (9.0)	Catechin	9.0	289	
x90		Fertaric acid, cis isomer*	9.2	325	
x94	x113 (9.6)	Caffeic acid	9.6	179	
x105		Astilbin	14.2	449	285, 151
	x114	Caffeic acid dimer*	9.6	357	135,86

Table S14: Single Ion Monitoring (SIM) acquisition parameters for analysis of PAC in Pinot Gris and Pinot Noir wines, Total Scan Cycle time = 500 ms

Compound	Mass (m/z)	MS2 Res	Dwell Time (ms)	Fragmentor	Cell Accelerator Voltage	Polarity
hexamers (PC)	1731.5	Unit (*)	16	135	5	Positive
c-hexamer (PC)	1729.5	Unit	16	135	5	Positive
5OH-pentamers (PD)	1523.4	Unit	16	135	5	Positive
c-5OH-pentamers (PD)	1521.4	Unit	16	135	5	Positive
4OH-pentamers (PD)	1507.4	Unit	16	135	5	Positive
c-4OH-pentamers (PD)	1505.4	Unit	16	135	5	Positive
3OH-pentamers (PD)	1491.4	Unit	16	135	5	Positive
c-3OH-pentamers (PD)	1489.4	Unit	16	135	5	Positive
2OH-pentamers (PD)	1475.4	Unit	16	135	5	Positive
c-2OH-pentamers (PD)	1473.4	Unit	16	135	5	Positive
OH-pentamers (PD)	1459.4	Unit	16	135	5	Positive
c-OH-pentamers (PD)	1457.4	Unit	20	135	5	Positive
pentamers (PC)	1443.4	Unit	16	135	5	Positive
c-pentamer (PC)	1441.4	Unit	50	135	5	Positive
4OH-tetramers (PD)	1219.3	Unit	16	135	5	Positive
c-4OH-tetramers (PD)	1217.3	Unit	16	135	5	Positive
3OH-tetramers (PD)	1203.3	Unit	50	135	5	Positive
c-3OH-tetramers (PD)	1201.3	Unit	16	135	5	Positive
2OH-tetramers (PD)	1187.3	Unit	50	135	5	Positive
c2OH-tetramers (PD)	1185.3	Unit	50	135	5	Positive
OH-tetramers (PD)	1171.3	Unit	50	135	5	Positive
c-OH-tetramer (PD)	1169.3	Unit	50	135	5	Positive
tetramers (PC)	1155.3	Unit	50	135	5	Positive
c-tetramer (PC)	1153.3	Unit	50	135	5	Positive
Trimers OH (PD)	883.2	Unit	16	135	5	Positive
Trimers (PC)	867.2	Unit	16	135	5	Positive
Dimers OH2 (PD)	611.1	Unit	16	135	5	Positive

Dimers OH (PD)	595.1	Unit	16	135	5	Positive
Dimers (PC)	579.1	Unit	16	135	5	Positive
PC = procyanidin		Unit(*) resolution = +/- 0.7 amu				
PD = prodelphinidin						

Table S15: Filtered PAC features observed in the LC-MS analysis for Pinot Gris

m/z	Retention Time	Compounds
579	18.2	Dimer (PC)
579	23.9	Dimer (PC)
595	24.4	Dimer OH (PD)
611	6.1	Dimers OH2 (PD)
611	6.4	Dimers OH2 (PD)
611	10.2	Dimers OH2 (PD)
611	11.8	Dimers OH2 (PD)
611	12	Dimers OH2 (PD)
611	14.2	Dimers OH2 (PD)
611	17.2	Dimers OH2 (PD)
611	18	Dimers OH2 (PD)
611	19	Dimers OH2 (PD)
611	20.1	Dimers OH2 (PD)
611	20.9	Dimers OH2 (PD)
611	21.8	Dimers OH2 (PD)
867	12.5	Trimer (PC)
867	15.8	Trimer (PC)
867	16.9	Trimer (PC)
867	18.7	Trimer (PC)
867	21.3	Trimer (PC)
883	7.3	Trimer OH (PD)
883	9.9	Trimer OH (PD)
883	11.5	Trimer OH (PD)
883	13.8	Trimer OH (PD)
883	16.7	Trimer OH (PD)
883	18	Trimer OH (PD)
1153	22.1	c-Tetramer (c-PC)
1153	22.7	c-Tetramer (c-PC)
1155	10.2	Tetramer (PC)
1155	17.2	Tetramer (PC)
1155	19.6	Tetramer (PC)
1169	17.1	c-OH-Tetramer (c-PD)
1171	10.1	OH-Tetramer (PD)
1171	10.7	OH-Tetramer (PD)

1171	11.5	OH-Tetramer (PD)
1171	14.5	OH-Tetramer (PD)
1171	15.2	OH-Tetramer (PD)
1171	15.9	OH-Tetramer (PD)
1171	17.1	OH-Tetramer (PD)
1171	18.6	OH-Tetramer (PD)
1171	18.7	OH-Tetramer (PD)
1171	23.8	OH-Tetramer (PD)
1171	24.7	OH-Tetramer (PD)
1441	24.7	c-Pentamer (PC)
1443	7.8	Pentamer (PC)
1443	11.2	Pentamer (PC)
1443	12.1	Pentamer (PC)
1443	13	Pentamer (PC)
1443	13.5	Pentamer (PC)
1443	14.6	Pentamer (PC)
1443	19	Pentamer (PC)
1443	22.2	Pentamer (PC)

Table S16: Filtered PAC features observed in the LC-MS analysis for Pinot Noir

m/z	Retention Time	Compounds
579	15.6	Dimer (PC)
579	35.4	Dimer (PC)
579	37.5	Dimer (PC)
579	39.7	Dimer (PC)
579	41.3	Dimer (PC)
579	47.6	Dimer (PC)
579	49.0	Dimer (PC)
579	17.3	Dimer (PC)
579	20.9	Dimer (PC)
579	22.3	Dimer (PC)
579	27.2	Dimer (PC)
579	29.2	Dimer (PC)
579	30.9	Dimer (PC)
579	33.8	Dimer (PC)
579	34.5	Dimer (PC)

595	15.4	Dimer OH (PD)
595	28.1	Dimer OH (PD)
595	29.5	Dimer OH (PD)
595	29.9	Dimer OH (PD)
595	30.3	Dimer OH (PD)
595	32.9	Dimer OH (PD)
595	33.6	Dimer OH (PD)
595	34.3	Dimer OH (PD)
595	35.2	Dimer OH (PD)
595	38.8	Dimer OH (PD)
595	40.6	Dimer OH (PD)
595	16.2	Dimer OH (PD)
595	41.2	Dimer OH (PD)
595	47.3	Dimer OH (PD)
595	49.4	Dimer OH (PD)
595	17.0	Dimer OH (PD)
595	20.2	Dimer OH (PD)
595	21.9	Dimer OH (PD)
595	24.1	Dimer OH (PD)
595	24.9	Dimer OH (PD)
595	26.4	Dimer OH (PD)
595	27.2	Dimer OH (PD)
611	10.4	Dimers OH2 (PD)
611	24.6	Dimers OH2 (PD)
611	35.4	Dimers OH2 (PD)
611	27.2	Dimers OH2 (PD)
611	28.2	Dimers OH2 (PD)
611	29.1	Dimers OH2 (PD)
611	30.2	Dimers OH2 (PD)
611	31.3	Dimers OH2 (PD)
611	38.6	Dimers OH2 (PD)
611	40.9	Dimers OH2 (PD)
611	41.1	Dimers OH2 (PD)
611	11.4	Dimers OH2 (PD)
611	42.4	Dimers OH2 (PD)
611	14.2	Dimers OH2 (PD)
611	15.4	Dimers OH2 (PD)

611	16.9	Dimers OH2 (PD)
611	19.9	Dimers OH2 (PD)
611	20.9	Dimers OH2 (PD)
611	21.5	Dimers OH2 (PD)
611	22.0	Dimers OH2 (PD)
867	13.7	Trimer (PC)
867	37.4	Trimer (PC)
867	38.4	Trimer (PC)
867	39.0	Trimer (PC)
867	39.6	Trimer (PC)
867	41.7	Trimer (PC)
867	41.7	Trimer (PC)
867	41.8	Trimer (PC)
867	42.7	Trimer (PC)
867	43.6	Trimer (PC)
867	20.8	Trimer (PC)
867	29.2	Trimer (PC)
867	31.6	Trimer (PC)
867	32.6	Trimer (PC)
867	33.4	Trimer (PC)
867	35.0	Trimer (PC)
867	36.1	Trimer (PC)
867	37.3	Trimer (PC)
883	9.3	Trimer OH (PD)
883	27.3	Trimer OH (PD)
883	27.3	Trimer OH (PD)
883	28.0	Trimer OH (PD)
883	28.4	Trimer OH (PD)
883	29.4	Trimer OH (PD)
883	29.9	Trimer OH (PD)
883	30.3	Trimer OH (PD)
883	30.7	Trimer OH (PD)
883	31.2	Trimer OH (PD)
883	33.0	Trimer OH (PD)
883	10.7	Trimer OH (PD)
883	33.9	Trimer OH (PD)
883	34.9	Trimer OH (PD)

883	37.0	Trimer OH (PD)
883	38.1	Trimer OH (PD)
883	38.6	Trimer OH (PD)
883	15.4	Trimer OH (PD)
883	17.5	Trimer OH (PD)
883	18.5	Trimer OH (PD)
883	19.2	Trimer OH (PD)
883	21.9	Trimer OH (PD)
883	24.7	Trimer OH (PD)
883	26.0	Trimer OH (PD)
1153	21.5	c-Tetramer (c-PC)
1155	16.2	Tetramer (PC)
1155	35.7	Tetramer (PC)
1155	36.2	Tetramer (PC)
1155	37.4	Tetramer (PC)
1155	37.5	Tetramer (PC)
1155	38.9	Tetramer (PC)
1155	38.9	Tetramer (PC)
1155	40.9	Tetramer (PC)
1155	42.4	Tetramer (PC)
1155	42.4	Tetramer (PC)
1155	44.6	Tetramer (PC)
1155	21.4	Tetramer (PC)
1155	27.6	Tetramer (PC)
1155	29.6	Tetramer (PC)
1155	31.0	Tetramer (PC)
1155	31.8	Tetramer (PC)
1155	32.9	Tetramer (PC)
1155	32.9	Tetramer (PC)
1155	33.4	Tetramer (PC)
1171	10.6	OH-Tetramer (PD)
1171	22.1	OH-Tetramer (PD)
1171	23.3	OH-Tetramer (PD)
1171	24.5	OH-Tetramer (PD)
1171	24.5	OH-Tetramer (PD)
1171	25.4	OH-Tetramer (PD)
1171	25.7	OH-Tetramer (PD)

1171	26.4	OH-Tetramer (PD)
1171	27.4	OH-Tetramer (PD)
1171	28.0	OH-Tetramer (PD)
1171	29.4	OH-Tetramer (PD)
1171	11.4	OH-Tetramer (PD)
1171	29.9	OH-Tetramer (PD)
1171	30.4	OH-Tetramer (PD)
1171	30.9	OH-Tetramer (PD)
1171	31.6	OH-Tetramer (PD)
1171	32.6	OH-Tetramer (PD)
1171	32.6	OH-Tetramer (PD)
1171	33.4	OH-Tetramer (PD)
1171	34.3	OH-Tetramer (PD)
1171	34.8	OH-Tetramer (PD)
1171	34.8	OH-Tetramer (PD)
1171	14.2	OH-Tetramer (PD)
1171	35.2	OH-Tetramer (PD)
1171	35.2	OH-Tetramer (PD)
1171	36.5	OH-Tetramer (PD)
1171	38.5	OH-Tetramer (PD)
1171	39.0	OH-Tetramer (PD)
1171	15.4	OH-Tetramer (PD)
1171	16.2	OH-Tetramer (PD)
1171	17.1	OH-Tetramer (PD)
1171	19.0	OH-Tetramer (PD)
1171	20.9	OH-Tetramer (PD)
1171	21.9	OH-Tetramer (PD)
1441	23.6	c-Pentamer (PC)
1443	23.6	Pentamer (PC)
1443	31.8	Pentamer (PC)
1443	32.6	Pentamer (PC)
1443	34.0	Pentamer (PC)
1443	35.4	Pentamer (PC)
1443	37.6	Pentamer (PC)
1443	41.3	Pentamer (PC)
1443	42.4	Pentamer (PC)

Table S17: Target identification of anthocyanins in Pinot Noir based on molecular ion [M-H] – (m/z) and lambda max (UV spectrum absorbance)

Anthocyanins	Retention time (+/- 0.1 min)	Molecular ion $[M]^+$ (m/z)	λmax (+/- 4mn)	Fragment Ion (in source) (m/z)
Delfinidin-3O-glucoside (Dl-3O- gl)	6.1	465	524	303
Petunidin-3-O-glucoside (Pt-3O-gl)	8.4	479	515	317
Peonidin-3-O-glucoside (Pn-3O-gl)	9.8	463	516	301
Malvidin-3-O-glucoside (Mv-3O-gl)	10.5	493	520	331
Malvidin-3-O-glucoside acetylated (Mv-3O-gluAc)	14.8	535	530	331

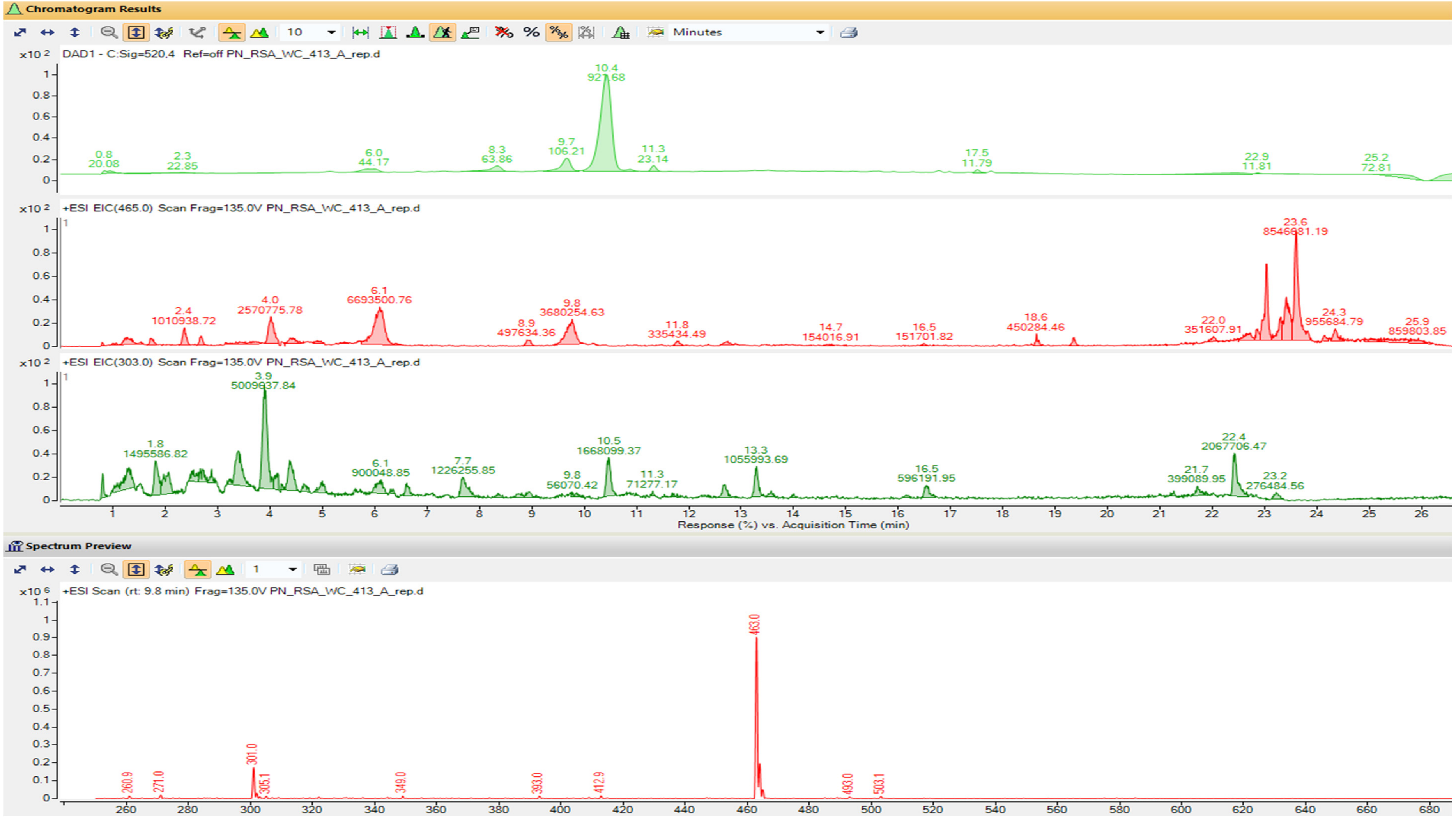

Figure S1: Example of a targeted analysis for peonidin-3-glucoside. DAD absorbance spectrum; EIC (Extracted Ion Chromatogram) targeting 463 m/z (precursor ion) and 303 m/z (product ion) at different retention times and MS2 fragments from ESI, reported as m/z ratio values respectively.

Table S18 : The variables importance in projection (VIP) in the PLS regression olfactory attribute "floral"aroma vs volatile compounds for Pinot Gris

Variable	VIP(1)	Standard deviation	Lower bound(95%)	Upper bound(95%)
VI	2.230	0.708	0.498	3.961
XXII	2.035	0.591	0.589	3.481
XXXI	1.874	0.587	0.438	3.310
XXXII	1.872	0.671	0.231	3.514
III	1.718	0.673	0.070	3.365
XIX	1.448	0.533	0.143	2.752
XXV	1.288	0.880	-0.864	3.441
XXXVII	1.284	0.701	-0.432	2.999
XX	1.181	0.486	-0.008	2.370
XXXV	1.086	0.728	-0.694	2.867
XXX	1.060	0.363	0.173	1.947
XVII	1.020	0.804	-0.948	2.987
XVIII	0.933	0.869	-1.194	3.060
XIII	0.916	0.933	-1.366	3.198
XII	0.885	0.918	-1.362	3.133
II	0.855	0.684	-0.819	2.528
XI	0.803	0.923	-1.456	3.062
XXI	0.682	0.515	-0.577	1.942
XXVI	0.675	0.564	-0.705	2.056
IV	0.639	0.460	-0.487	1.766

XXXIII	0.615	0.647	-0.968	2.198
XXXIV	0.597	0.613	-0.903	2.096
XV	0.567	0.785	-1.354	2.487
I	0.547	0.359	-0.330	1.425
X	0.485	0.290	-0.224	1.195
IX	0.475	0.420	-0.554	1.503
XXIII	0.461	0.583	-0.966	1.888
VII	0.378	0.321	-0.408	1.165
XVI	0.366	0.277	-0.311	1.043
XXVII	0.326	0.517	-0.940	1.592
XIV	0.317	0.491	-0.885	1.518
XXIV	0.157	0.512	-1.097	1.411
XXXVIII	0.146	0.480	-1.027	1.320
XXVIII	0.119	0.284	-0.575	0.813
XXIX	0.065	0.338	-0.763	0.893
VIII	0.048	0.230	-0.515	0.611
V	0.022	0.331	-0.789	0.832

Table S19: The variables importance in projection (VIP) in the PLS regression olfactory attribute "green bell pepper" aroma vs volatile compounds for Pinot Noir

Variable	VIP(2)	Standard deviation	Lower bound(95%)	Upper bound(95%)
XXXIII	1.533	0.736	-0.086	3.153
III	1.486	0.537	0.304	2.668
XXVIII	1.204	0.616	-0.151	2.559
XLIII	1.142	0.842	-0.711	2.995
XLI	1.060	0.316	0.365	1.754
X	0.993	0.303	0.326	1.660
VIII	0.991	0.175	0.607	1.375
XLII	0.985	0.190	0.567	1.402
XXI	0.940	0.329	0.215	1.665
VI	0.918	0.242	0.385	1.450
XVI	0.915	0.430	-0.031	1.861
XXXVIII	0.895	0.203	0.449	1.341
XL	0.853	0.385	0.004	1.701
XXIV	0.847	0.223	0.357	1.337
XLVII	0.821	0.632	-0.569	2.211
XIV	0.679	0.623	-0.692	2.050
XXX	0.589	0.549	-0.619	1.797
XLIV	0.578	0.586	-0.713	1.868

Table S20: The variables importance in projection (VIP) in the PLS regression olfactory attribute “cherry” aroma vs volatile compounds for Pinot Noir

Variable	VIP(2)	Standard deviation	Lower bound(95%)	Upper bound(95%)
XXVIII	1.966	0.854	0.087	3.844
VI	1.427	0.295	0.777	2.077
III	1.179	0.527	0.018	2.340
XLII	1.148	0.562	-0.089	2.385
XVI	1.147	0.651	-0.286	2.580
VIII	1.131	0.480	0.075	2.188
XXI	1.080	0.347	0.316	1.844
XXX	1.063	0.482	0.004	2.123
XLI	1.058	0.381	0.221	1.896
XL	1.032	0.263	0.453	1.610
X	0.918	0.249	0.370	1.466
XLVII	0.735	1.098	-1.681	3.151
XXXVIII	0.663	0.226	0.166	1.161
XLIII	0.432	0.597	-0.882	1.745
XXXIII	0.325	0.301	-0.336	0.987
XIV	0.268	0.552	-0.947	1.484
XLIV	0.261	0.639	-1.145	1.666
XXIV	0.249	0.598	-1.068	1.565

Table S21: The variables importance in projection (VIP) in the PLS regression olfactory attribute "licorice" aroma vs volatile compounds for Pinot Noir

Variable	VIP(2)	Standard deviation	Lower bound(95%)	Upper bound(95%)
XLIII	1.596	0.365	0.792	2.399
XLIV	1.249	0.942	-0.825	3.323
XIV	1.188	1.010	-1.035	3.411
XXIV	1.182	0.448	0.197	2.167
XLI	1.154	0.360	0.363	1.946
XVI	1.113	0.347	0.350	1.876
XXI	1.096	0.507	-0.020	2.211
XXX	1.080	0.270	0.487	1.674
XLVII	1.059	0.996	-1.134	3.252
XLII	1.037	0.392	0.175	1.899
XL	0.974	0.380	0.137	1.811
X	0.905	0.367	0.097	1.713
VIII	0.846	0.481	-0.212	1.903
VI	0.815	0.596	-0.497	2.127
III	0.638	0.490	-0.440	1.717
XXVIII	0.456	0.439	-0.510	1.422
XXXVIII	0.307	0.312	-0.379	0.993
XXXIII	0.307	0.471	-0.730	1.343

Table S22: The variables importance in projection (VIP) in the PLS regression olfactory attribute "fresh wood" aroma vs volatile compounds for Pinot Noir

Variable	VIP(3)	Standard deviation	Lower bound(95%)	Upper bound(95%)
XXVIII	1.817	0.908	-0.181	3.815
XXIV	1.295	0.316	0.599	1.991
XLIII	1.213	0.381	0.375	2.051
XIV	1.091	0.639	-0.315	2.496
III	1.051	0.274	0.448	1.654
XLIV	1.036	0.658	-0.413	2.485
XLVII	1.005	0.513	-0.124	2.134
XXX	1.002	0.211	0.538	1.466
XXXVIII	0.984	0.241	0.453	1.515
XXXIII	0.963	0.295	0.313	1.614
VIII	0.834	0.192	0.413	1.256
XLII	0.811	0.189	0.394	1.228
XL	0.798	0.312	0.112	1.484
XLI	0.778	0.221	0.291	1.264
XXI	0.717	0.434	-0.240	1.673
XVI	0.709	0.314	0.017	1.401
VI	0.615	0.193	0.190	1.041
X	0.530	0.144	0.212	0.848
